# Clozapine-dependent inhibition of EGF/neuregulin receptor (ErbB) kinases

**DOI:** 10.1038/s41398-019-0519-1

**Published:** 2019-08-01

**Authors:** Yutaro Kobayashi, Yuriko Iwakura, Hidekazu Sotoyama, Eiko Kitayama, Nobuyuki Takei, Toshiyuki Someya, Hiroyuki Nawa

**Affiliations:** 1 Department of Neurobiology, Brain Research Institute, Niigata University, 1-757 Asahimachi, Chuo-ku Niigata, 951-8585 Japan; 2 Department of Psychiatry, Graduate School of Medical and Dental Sciences, Niigata University, 1-757, Asahimachi-dori, Niigata, 951-8510 Japan

**Keywords:** Pharmacology, Molecular neuroscience

## Abstract

Clozapine is an antipsychotic agent prescribed to psychotic patients exhibiting tolerance and/or resistance to the conventional antipsychotic medications that mainly drive monoamine antagonism. As the pharmacological fundamentals of its unique antipsychotic profile have been unrevealed, here, we attempted to obtain hints at this question. Here, we found that clozapine directly acts on ErbB kinases to downregulate epidermal growth factor (EGF)/neuregulin signaling. In cultured cell lines and cortical neurons, EGF-triggered ErbB1 phosphorylation was diminished by 30 μM clozapine, but not haloperidol, risperidone, or olanzapine. The neuregulin-1-triggered ErbB4 phosphorylation was attenuated by 10 μM clozapine and 30 μM haloperidol. We assumed that clozapine may directly interact with the ErbB tyrosine kinases and affect their enzyme activity. To test this assumption, we performed in vitro kinase assays using recombinant truncated ErbB kinases. Clozapine (3–30 μM) significantly decreased the enzyme activity of the truncated ErbB1, B2, and B4 kinases. Acute in vivo administration of clozapine (20 mg/kg) to adult rats significantly suppressed the basal phosphorylation levels of ErbB4 in the brain, although we failed to detect effects on basal ErbB1 phosphorylation. Altogether with the previous findings that quinazoline inhibitors for ErbB kinases harbor antipsychotic potential in animal models for schizophrenia, our present observations suggest the possibility that the micromolar concentrations of clozapine can attenuate the activity of ErbB receptor kinases, which might illustrate a part of its unique antipsychotic psychopharmacology.

## Introduction

Clozapine was synthesized in 1958 as a new generation antipsychotic drug^1^. Clozapine exhibits a unique antipsychotic profile represented by its pharmacological efficacy on the negative symptoms and reduced extra pyramidal effects^1–3^. In contrast, typical antipsychotic agents such as haloperidol, fluphenazine, and phenothiazines derivatives were developed to block dopamine neurotransmission but produce extra pyramidal effects and weight gain^4,5^. The discovery of clozapine has led to the development of atypical antipsychotic drugs such as risperidone, olanzapine, and quetiapine^4,6,7^. Unlike other atypical antipsychotic agents, clozapine exhibits the unique antipsychotic pharmacology of suicide prevention and is applicable to the patients exhibiting tolerance to the conventional antipsychotic drugs^7,8^. In spite of these favorable effects of clozapine, the prescription of clozapine is highly regulated owing to severe side effects such as agranulocytosis and myocarditis^9–12^. The unconventional characteristics of clozapine are presumably attributed to neither dopamine or serotonin antagonism and thus, the molecular mechanisms underlying this unique pharmacology remain to be characterized^13–15^.

Several antipsychotic compounds including chlorpromazine, aripiprazole, and clozapine possess biological activities that cannot be illustrated by their anti-monoaminergic actions^16–18^. For example, chlorpromazine and thioridazine produce exocytotoxic influences on various cancer cells, recruiting antiproliferative signaling or autophagy cascades^19,20^. Aripiprazole is reported to exert anti-cancer actions, inhibiting Src kinase and PI3 kinase^21^. However, the kinase-inhibiting activities of antipsychotic compounds appear to be controversial especially in in vivo conditions. Haloperidol and clozapine act on neurons to induce neurite extension, stimulating Ras-MAP kinase signaling^22–24^. Treatment with several antipsychotics results in the increases in Akt signaling in the brain^25–28^. These reports propose the possibility that several antipsychotic compounds such as clozapine carry unrevealed biological activities on various protein kinases. However, it is poorly understood whether these novel activities of antipsychotic agents can be ascribed to their monoaminergic function or novel chemical actions not involving neurotransmission^29^.

The upregulation of epidermal growth factor (EGF) and/or neuregulin signaling has been implicated in the neuropathology of schizophrenia, as well as in its animal modeling^30,31^. According to this hypothesis, we previously tested the antipsychotic potential of various kinase inhibitors for epidermal growth factor receptor family (ErbB) and found that these kinase inhibitors can ameliorate the behavioral deficits of several animal models for schizophrenia^32–34^. The present experiments were designed on the basis of the hypothesis that ErbB kinases might be potential molecular targets of clozapine.

Employing cancer cells as well as primary cultured neurons, we compared the effects of various antipsychotic compounds on cell growth/survival and explored the molecular targets of clozapine, focusing on their actions on EGF/neuregulin receptor kinases (i.e., ErbB1/2/4)^30,31^. In particular, the use of the non-neuronal cancer cell lines that lack neurotransmitter synthesis and release allowed us to minimize the anti-monoaminergic actions of antipsychotic drugs in our assays. Among many antipsychotic drugs, we selected olanzapine, haloperidol, and risperidone as control compounds, all of which have been well characterized with respect to their receptor pharmacology and neurobiology. Based on the obtained results in culture and in vitro, we attempted to illustrate the unique antipsychotic actions and the side effects of clozapine on schizophrenic patients.

## Materials And methods

### Antipsychotic agents

The following four antipsychotic compounds were used: olanzapine and haloperidol (both Wako chemicals Inc., Osaka, Japan), clozapine (Adooq Bioscience, California, USA), and risperidone (Risperdal solution; Janssen Pharma, Belgium). All antipsychotic compounds except risperidone were dissolved in dimethyl sulfoxide (DMSO). Alternatively, antipsychotic compounds were dissolved in Triton X-100 in the in vitro kinase assay.

### Cell culture

Human astroglioma cells (U87MG) were obtained from the Health Protection Agency (Culture Collections, Salisbury, UK). Human epidermoid cells (A431) were obtained from the RIKEN Cell Bank (Ibaraki, Japan). Human breast cancer cells (MDA-MB-453) was a kind gift from Dr. Higashiyama^35,36^. Cells were grown in DMEM or RPMI-1640 containing 10% fetal bovine serum and kanamycin (50 μg/mL). Cancer cells were plated at a cell density of 10^3^ onto the 96-well culture plates and grown for 24 h. Cell growth/survival for the following 48 h was measured using the Cell Counting Kit-8 (Dojindo, Kumamoto, Japan). For phosphorylation assays, cells were starved in serum-free DMEM for 10–12 h and then treated with 0–100 µM of the antipsychotic compounds for 20–30 min followed by the stimulation of EGF or neuregulin-1 (30 ng/mL, Peprotech Inc, Ehovot, Israel) for 5 min. The used neuregulin-1 was a core EGF domain peptide whose amino acid sequence is shared by all splice variants of neuregulin-1^30^

Whole cerebral neocortices of fetal rats (Sprague-Dawley, embryonic day 18–19, male and female) were enzymatically dissociated, plated on 35 mm-diameter dishes at a density of 1.5 × 10^6^ cells/dish, and grown in the serum-free condition as described previously^37^. Cortical cultures were maintained for 5 days and subjected to treatment with EGF or neuregulin-1 as described above.

### In vivo administration of clozapine

Clozapine was dissolved in sodium-citrate buffer (pH 5.5) and intraperitoneally administered to adult rats (Sprague-Dawley, 8–9 weeks all male, SLC Japan, Hamamatsu, Japan) at the dose of 20 mg/kg. One hour after administration, rats were euthanized and subjected to brain dissection. The brain regions such as hippocampus and frontal cortex were obtained and frozen in dry ice. The treatment of these animals was in accordance with the local and international guidelines on the ethical use of laboratory animals. All animal procedures adopted in this study were approved by and conducted under the control of the Niigata University Animal Care and Use Committee.

Randomization and blinding of individual rats were not applied to this animal experiment.

### Western blotting

Cultured cells or frozen tissue were homogenized with the lysis buffer containing 1% lithium dodecyl sulfate, 50 mM Tris (pH 6.8), 500 µM EDTA, 1 mM NaF, 1 mM Na_2_VO_4_, and protease inhibitor cocktail (Complete mini; Roche), and 1 mM phenylmethylsulfonyl fluoride (PMSF). Cell lysates were heat-denatured as described previously^36,37^. Protein concentrations were determined using a Micro BCA kit (Thermo Fisher Scientific, Massachusetts, USA). Typically, protein samples (20 µg/lane) were loaded and separated by sodium dodecyl sulfate-polyacrylamide gel electrophoresis. Proteins were transferred to a polyvinylidene difluoride membrane (PVDF) by electrophoresis in transfer buffer^36,37^. The membranes were incubated overnight with the indicated primary antibodies at 4 °C. The dilution ratio of the primary antibodies was 1:1000 unless specified below. Membranes were rinsed with the wash buffer (TBST; 25 mM Tris pH 7.4, 137 mM NaCl, 2.68 mM KCl, and 0.1% Tween-20) and subsequently incubated with peroxidase-conjugated secondary antibodies (1:10,000, Agilent Technologies, California, USA). The immunoreactivity was visualized with a chemiluminescence reaction using Western Lightning Plus-ECL (PerkinElmer, Massachusetts, USA) or ImmunoStar LD (Wako) as a substrate. Chemiluminescence images were taken at various time points by a 16-bit CCD camera system, G:Box Chemi XRQ (Syngene, Cambridge, UK). The intensity of the immunoreactive band was normalized to the background signal and automatically quantitated with an image analysis software, GeneTools (Syngene).

The primary antibodies against phospho-Ser473-Akt (1:2000, Cat#4060), Akt (1:2000, Cat#9272), phospho-Thr 202/Tyr 204-Erk1/2 (1:1000, Cat#4370), Erk1/2 (1:1000, Cat#4695), phospho-Tyr845-ErbB1 (1:1000, Cat#2231), phospho-Tyr1045-ErbB1 (1:1000, Cat#2237), ErbB1 (1:1000, Cat#2232), phospho-Tyr1196-ErbB2 (1:1000, Cat#6942), phospho-Tyr1248-ErbB2 (1:10,000, Cat#2247), phospho-Tyr1197-ErbB3 (1:1000, Cat#4561), phospho-Tyr1289-ErbB3 (1:500, Cat#4791), and phospho-Tyr1284-ErbB4 (1:1000, Cat#4757) were obtained from Cell Signaling (Massachusetts, USA)^32,38^. Additionally, the following primary antibodies were used; anti-phospho-Tyr1139-ErbB2 (1:1000, Cat#1991, Eptomics, Curlingame, CA USA), anti-ErbB2 (1:1000, Cat#2064, Eptomics Abcam), anti-phospho-Tyr1328-ErbB3 (1:1000, Cat#135654, Santa Cruz Biotechnology, Texas, USA), phospho-Tyr1173-ErbB1 (1:1000, Cat#sc-12351, Santa Cruz), anti-ErbB3 (1:1000, Cat#1186, Eptomics), anti-phospho-Tyr1056-ErbB4 (1:1000, Cat#13094, Bioss, Boston, USA), anti-phospho-Tyr1242-ErbB4 (1:1000, Cat#12727, Signalway Antibody, Maryland, USA), and anti-ErbB4 (1:1000, Cat#2218, Eptomics)^36,39^. No randomization or blinding was used for sample loading.

### In vitro kinase assay

A 96-well plate was precoated with tyrosine-glutamate heteropolymer (80 µg/mL, Merck) followed by a non-protein membrane blocking agent, Can Gel Signal Block (Toyobo Biochemical Inc., Osaka Japan). After washing, the recombinant enzyme, namely, truncated ErbB1 kinase (7.1 ng/mL, SignalChem, British Columbia, Canada), truncated ErbB2 kinase (60 ng/mL, Thermo Fisherc), or truncated ErbB4 kinase (6.0 ng/mL, Thermo Fisher) was added to the wells along with the kinase reaction buffer (100 µL) comprising 0.2% Triton X-100, 40 mM Tris HCl (pH 7.5), 5 mM β-glycerophosphate, 0.1 mM Na_3_VO_4_, 10 mM MgCl_2_, and 5 mM MnCl_2_. The enzyme stabilizer dithiothreitol was not further supplemented in addition to the carryover of dithiothreitol present in the kinase stock solution. It was presumed that this strong reducing agent could chemically react with or inhibit the action of the antipsychotic compounds used^40^. The tyrosine kinase reaction was initiated with the addition of 4 mM ATP, maintained at 37 °C for 30 min and terminated with a stopping buffer containing 10 mM EDTA, 1% bovine serum albumin, and 0.5% gelatin. Tyrosine-phosphorylation of the substrate was detected by a peroxide-conjugated anti-phosphotyrosine antibody (1:500, Cat#5465S, Cell Signaling). The trapped immunoreactivity was measured with a peroxidase substrate mixture (521 mM tetramethylbenzidine, 0.005% H_2_O_2_ and 130 mM sodium-citrate buffer, pH 8.0) at 37 °C for 15 min and terminated by adding 0.3 M phosphoric acid. The optical absorbance was measured at 450 nm and compared with that of the standard reactions obtained with fixed amounts of the recombinant ErbB kinase. No randomization or blinding was used.

### Statistical design

As our preliminary studies found marked effects of clozapine on ErbB kinase activity, the present study focused on such large effects of clozapine (i.e., effect sizes; *d* > 0.8 and *η*^2^ > 0.14), which can be implicated biologically and pharmacologically. For this purpose, we set-up the minimum number of samples (*n* = 3–6), lowering the type I error level (*α*) but not minimizing the type II error level (*β*). With this respect, some experimental data sets, which carried the lower statistical power (i.e., 1 – *β* < 0.8), are indicated in figure legends to warn about their type II error risk. Kolmogolov–Smirnov test was applied to individual data sets (*n* > 3) and failed to reject the null hypothesis that the data variation fits the normal distribution (data not shown). Accordingly, all data were subjected to parametric analyses and are expressed as means ± SEM. Brown–Forsythe test failed to reject the homogeniety of data variance in almost all the experiments as indicated in figure legends. When this test rejected the null hypothesis for the data variance, those data were directly analyzed by planned multiple comparisons of Welch *t-*test (two tails) with Bonferroni’s compensation. When univariate data with more than two groups harbored similar data distributions, those were subjected to analysis of variance (ANOVA) to assess the statistical differences among the experimental groups and among doses, followed by a Tukey’s post-hoc test for multiple comparisons. Alternatively, Student *t-*test or Welch *t-*test (both two tails) was applied to univariate data from two groups. Statistical analyses were performed using SPSS software (version 11.0J, SPSS Japan Tokyo, Japan). A *P-*value of less than the *α* level (0.05/N) was considered as statistically significant (*N*; the number of the planed comparisons for Bonferroni’s compensation or *N* = 1 for the other tests).

## Results

### Effects of antipsychotic compounds on non-neuronal cancer cells in culture

**Fig. 1:**
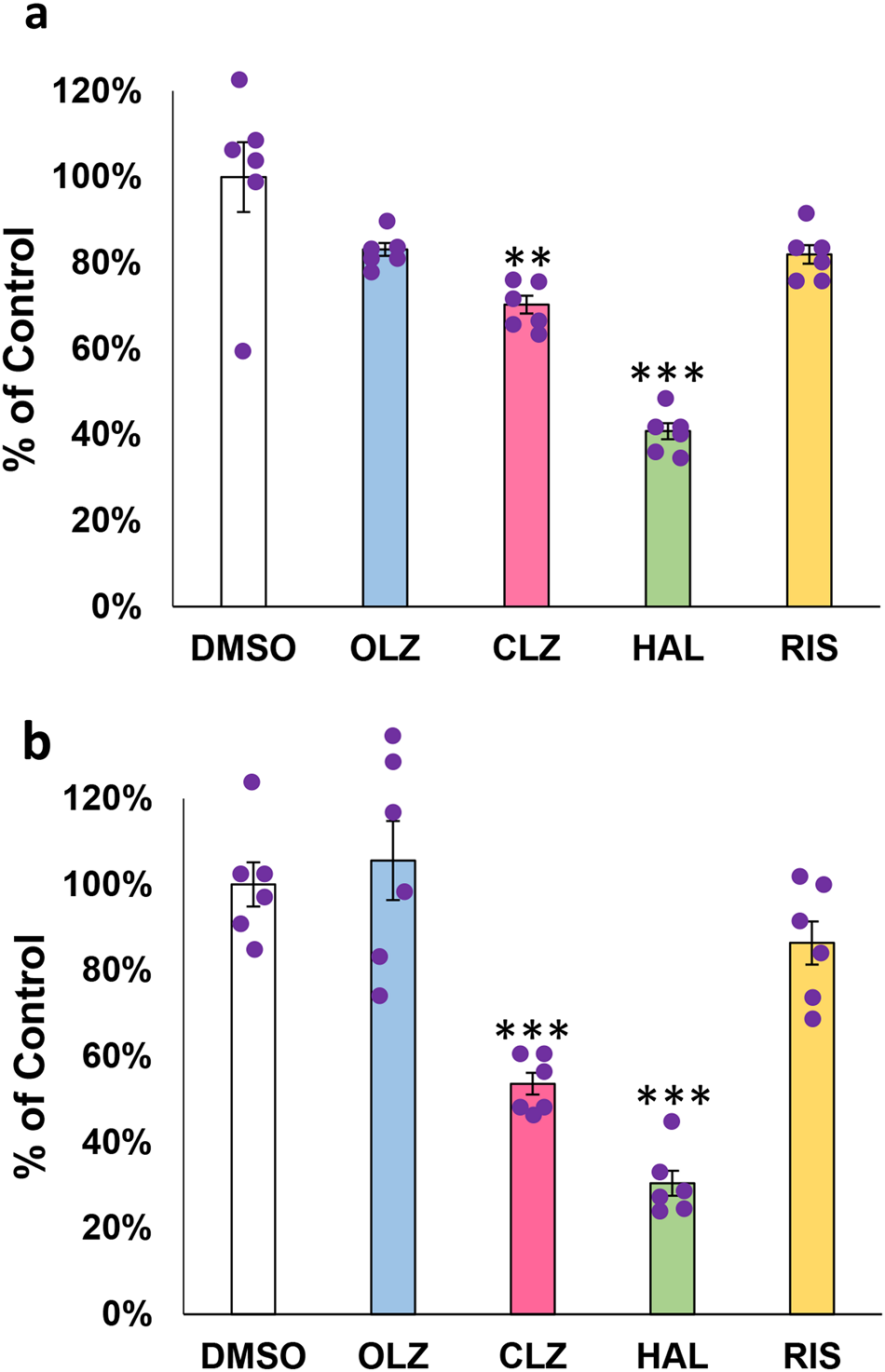
Effects of antipsychotic compounds on the growth/survival of cancer cells in culture. Treatment with 0.1% DMSO (vehicle control), 30 µM olanzapine (OLZ), 30 µM clozapine (CLZ), 30 µM haloperidol (HAL), or 30 µM risperidone (RIS) for 48 h affected the proliferation/survival of U87MG cells (**a**) and A431 cells (**b**) grown in the normal growth medium. The number of living cells was estimated using the CCK-8 cell counting kit before and after the drug treatment. The graph reveals % ratio to the final cell number of control cultures treated with DMSO alone (mean ± SE, *n* = 6 sister cultures). Brown–Forsythe test assessed the homogeneity of data variance in **a** (*P* = 0.227), but not in **b** (*P* = 0.075). One-way ANOVA followed by Tukey’s test was applied to **a**; **P < 0.01 and ***P < 0.001 vs. control culture (DMSO). *β* < 0.001 and *η*^2^ = 0.799 for **a**. Multiple comparisons of Welch *t*-test with Bonferroni’s compensa*t*ion was performed with the given difference in data variance among groups in **b**; **P < 0.01/4 and ***P < 0.001/4 vs. control culture (DMSO). *β* = 0.923, 0.000, 0.000, and 0.600; *d* = 0.307, −4.662, −6.799, and −1.090 for OLZ, CLZ, HAL, and RIS vs. DMSO, respectively

Various antipsychotic compounds are known to attenuate cancer cell survival and proliferation^19–24^. We estimated the contribution of ErbB signaling to the growth/survival of cancer cell lines, human astroglioma U87MG and human epidermoid carcinoma A431, which are reported to express high levels of ErbB kinases to regulate their cell growth and survival^41–44^. In this primary assessment, we applied the antipsychotic agent (30 µM) under investigation, namely, olanzapine, clozapine, haloperidol, or risperidone to U87MG and A431 cultures (Fig. 1). The application of clozapine and haloperidol to normal growth media significantly inhibited cell growth as monitored by the cell counts of U87MG and A431 [*F*(4, 25) = 24.86, *P* < 0.001 for U87MG; *F*(4, 25) = 28.42, *P* < 0.001 for A431]. The growth inhibitory effect of clozapine on those cells was dose-dependent (Supplemental Fig. S1). These results suggest the possibility that clozapine may act on EGF or other growth factor signaling to attenuate cell proliferation and/or survival,

### Inhibitory effects of antipsychotic compounds on ErbB receptor phosphorylation in culture

**Fig. 2:**
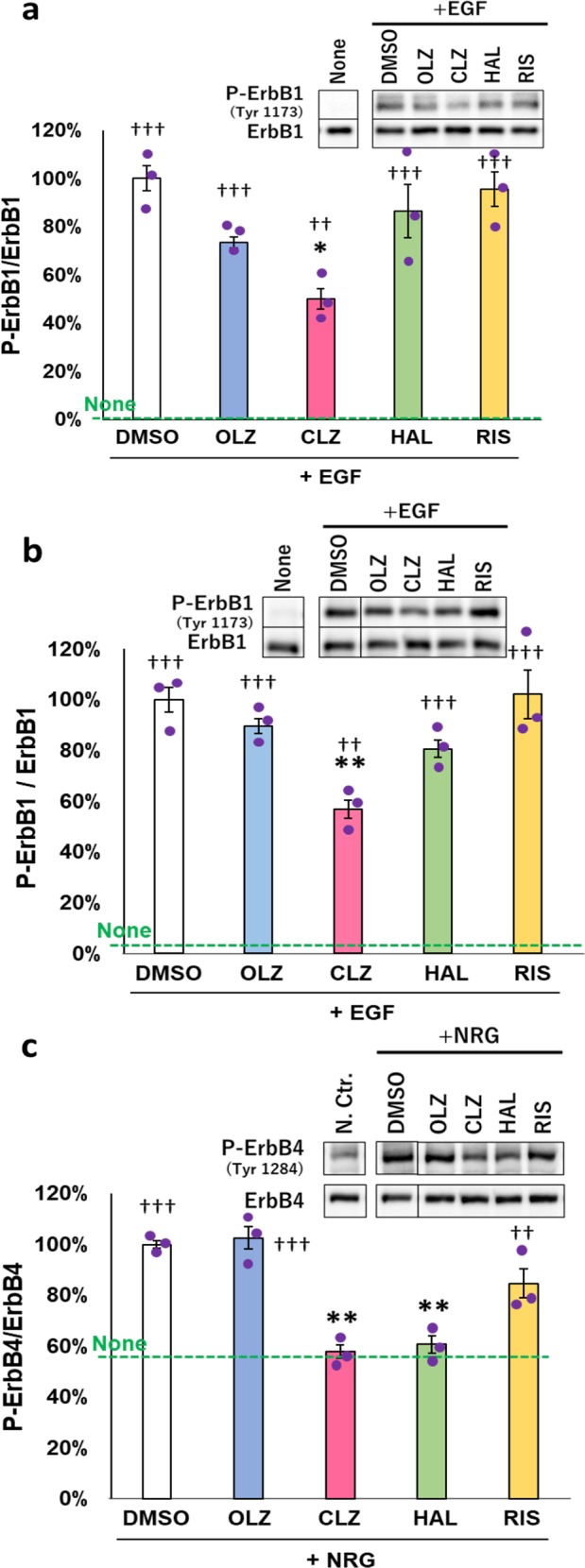
Effects of antipsychotic compounds on phosphorylation of ErbB1 and ErbB4. Inhibition of EGF-induced ErbB1 phosphorylation by antipsychotic compounds in U87MG cells (**a**) and cultured cortical neurons (**b**) was determined by immunoblotting for phosphorylated (P-ErbB1) and total ErbB1 (ErbB1). **c** The inhibition of neuregulin-1 (NRG)-induced ErbB4 phosphorylation by antipsychotic compounds in cultured cortical neurons was similarly determined by immunoblotting. Typical immunoblots for phosphorylated and total ErbB4 are shown. Bar represents the ratio to the EGF-induced or neuregulin-1-induced phosphorylation levels (100% maximum) obtained in the absence of antipsychotic compounds (mean ± SE, *n* = 3 sister cultures). Horizontal broken lines mark the averaged basal phosphorylation level of ErbB1 (None; 0.6 ± 0.12% for U87MG; 3.5 ± 0.7% for cortical neurons) and that of ErbB4 in control cultures (None; 54.0 ± 4.6%). The abbreviations of the antipsychotic compounds are same as indicated in Fig. 1. *P < 0.05 and **P < 0.01 vs. positive control cultures (100%; DMSO), ^††^*P* < 0.01 and ^†††^*P* < 0.001 vs. negative control cultures (None), one-way ANOVA followed by Tukey’s test. *β* = 0.104 and *η*^2^ = 0.710 for **a**, *β* = 0.051 and *η*^2^ = 0.750 for **b**, *β* < 0.001 and *η*^2^ = 0.894 for **c**. Brown–Forsythe test suggested the homogeneity of data variance; *P* = 0.515 for **a**, 0.829 for **b**, and 0.844 for **c**. NRG; neuregulin-1

To investigate the acute influence of the antipsychotic compounds on ErbB signaling, we examined the effects of these compounds on ErbB phosphorylation. Following the pretreatment with an antipsychotic compound (30 μM), ErbB1 phosphorylation was triggered by EGF application to cultured U87MG cells, as well as to rat cortical neurons (Fig. 2). Clozapine, but not olanzapine, haloperidol, or risperidone, significantly attenuated ErbB1 phosphorylation (Tyr1173) in U87MG cells (Fig. 2a) [*F*(4, 10) = 6.127, *P* = 0.009]. Similarly, in cultured cortical neurons, treatment with 30 µM clozapine significantly attenuated ErbB1 phosphorylation (Tyr1173) (Fig. 2b) [*F*(4, 10) = 7.480, *P* = 0.005]. The phosphorylation of ErbB4 (Tyr1284), which was induced by application of neuregulin-1, was attenuated by clozapine and haloperidol (30 µM each) [*F*(4,10) = 20.99, *P* < 0.001] but not by olanzapine or risperidone (Fig. 2c).

We also investigated the effects of antipsychotic compounds alone on basal phosphorylation levels of ErbB1 and ErbB4, using cultured cortical neurons (Supplementary Fig. S2). All compounds had no significant influence on ErbB1 phosphorylation (Tyr1173) even at the dose of 100 µM [*F*(4,10) = 0.635, *P* = 0.649]. In contrast, the basal phosphorylation of ErbB4 (Tyr1284) was diminished by clozapine and haloperidol as seen in the above ligand-stimulated condition [*F*(4,10) = 18.951, *P* < 0.001].

Ligand interaction leads to the dimerization of the ErbB receptor tyrosine kinases, followed by trans-phosphorylation of various tyrosine residues of the partner ErbB molecules as substrates^30,31,45,46^. Our preliminary experiments suggest that clozapine appeared to similarly suppress the ligand-induced phosphorylation of all the tyrosine residues of ErbB1/2/4 receptors except those of ErbB3 (Supplementary Fig. S3). For example, the inhibitory effects of clozapine on phosphorylation levels of distinct ErbB4 tyrosine residues (Try1056, Tyr1242, and Tyr1284) were indistinguishable (*F*(5,12) = 12.10, *P* < 0.001 for main effects and *F*(2,12) = 1.839, *P* = 0.201 for their interaction).

### Clozapine-dose-dependent inhibition of EGF- or neuregulin-induced ErbB phosphorylation and their downstream signaling

**Fig. 3:**
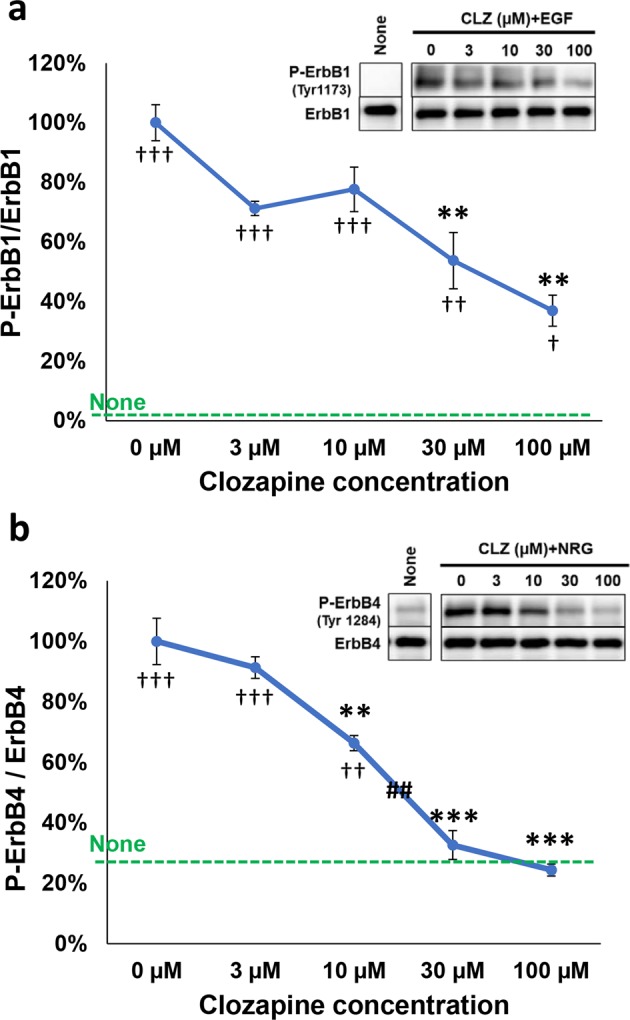
Dose-dependent inhibition of the ligand-induced ErbB1/4 phosphorylation by clozapine. **a** Phosphorylation levels of ErbB1 in U87MG cells treated with 0–100 µM clozapine (CLZ) are presented. **b** Phosphorylation levels of ErbB4 in cultured cortical neurons pretreated with 0–100 µM clozapine are presented. The graph reveals % ratio to the EGF-induced or neuregulin-1-induced phosphorylation levels (100% maximum) (mean ± SE, *n* = 3 sister cultures). Horizontal broken lines mark the averaged basal phosphorylation level of ErbB1 (None; 1.1 ± 0.7%) or that of ErbB4 in untreated cultures (None; 27.0 ± 3.3%). *P < 0.05, **P < 0.01, and ***P < 0.001 vs. control cultures (0 μM clozapine), ^†^*P* < 0.05, ^††^*P* < 0.01, and ^†††^*P* < 0.001 vs. negative control cultures (None), ^##^*P* < 0.01 between adjacent concentrations, one-way ANOVA followed by Tukey’s test. *β* = 0.023 and *η*^2^ = 0.781 for **a**, *β* < 0.001 and *η*^2^ = 0.936 for **b**. Brown–Forsythe test suggested the homogeneity of data variance; *P* = 0.829 for **a** and 0.788 for **b**. NRG1; neuregulin-1

Individual phosphotyrosine residues of ErbB kinases recruit distinct signal transducers such as Grb2 and Shc, leading to Erk and Akt signal transduction^31,45^. We examined the dose effects of clozapine on the EGF-triggered ErbB1 phosphorylation as well as neuregulin-induced ErbB4 phosphorylation in cultured U87MG cells and cortical neurons, respectively. In U87MG cells, a reduction in ErbB1 phosphorylation was dose-dependent [*F*(4, 10) = 8.93, *P* = 0.002] (Fig. 3a). The minimum concentration of clozapine required for significant inhibition of ErbB1 phosphorylation was 30 μM. A dose-dependent decrease in the ErbB4 phosphorylation was also observed in cultured neurons [*F*(4, 10) = 36.69, *P* < 0.001] (Fig. 3b). The lowest concentration of clozapine that significantly suppressed ErbB4 phosphorylation was 10 μM. The half maximal inhibitory concentration (IC_50_) of clozapine was ~47 µM for Tyr1173-phosphorylation of ErbB1 and ~16 µM for Tyr1284-phosphorylation of ErbB4. The inhibitory action of clozapine on ErbB1 phosphorylation was more modest in A431 cells, however (Supplemental Fig. S4).

**Fig. 4:**
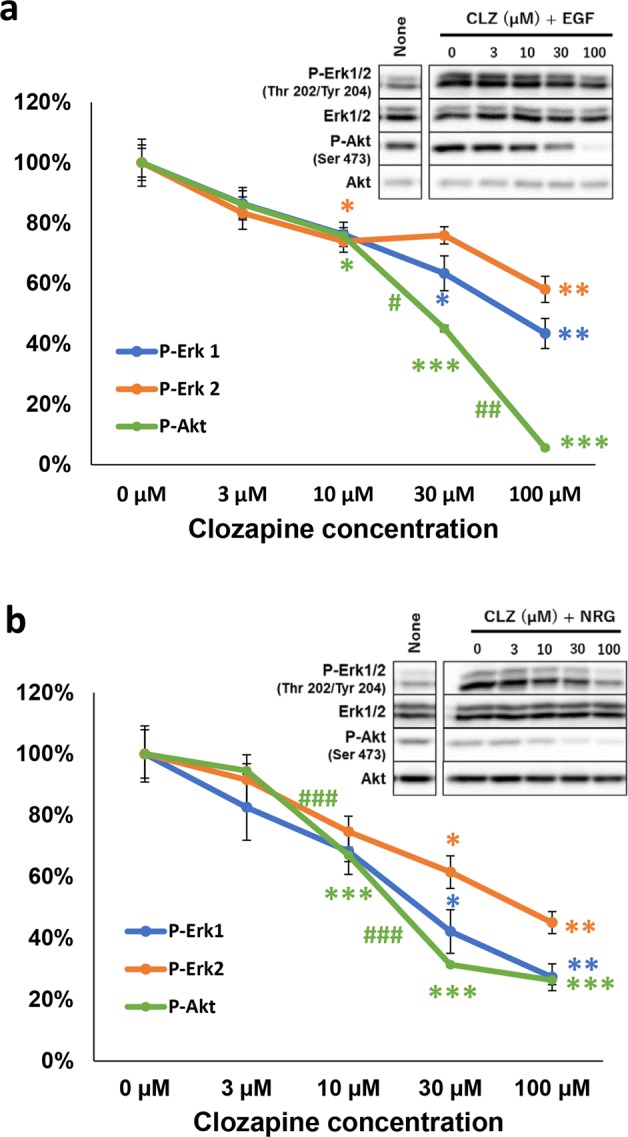
Effects of clozapine doses on the inhibition of ErbB downstream signaling. **a** Dose effects of clozapine (0–100 µM) on the phosphorylation of Erk1, Erk2, and Akt in U87MG cells were determined by immunoblotting and normalized with total Erk1/2 and total Akt levels in the samples. **b** In cultured cortical neurons, dose effects of clozapine on the phosphorylation of Erk1, Erk2, and Akt are presented. The graphs reveal % ratio (mean ± SE, *n* = 3 sister cultures) to their maximum levels of the ligand-stimulated cultures obtained in the absence of clozapine (0 μM clozapine). Typical immunoblots are shown. *P < 0.05, **P < 0.01, and ***P < 0.001 vs. control culture (0 μM clozapine), ^#^*P* < 0.05, ^##^*P* < 0.01, and ^###^*P* < 0.001 between adjacent concentrations, one-way ANOVA followed by Tukey’s test. In **a** *β* = 0.003 and *η*^2^ = 0.833 for P-Erk1/Erk1, *β* = 0.051 and *η*^2^ = 0.750 for P-Erk2/Erk^2^, *β* < 0.001 and *η*^2^ = 0.955 for P-Akt/Akt. In **b** *β* = 0.023 and *η*^2^ = 0.781 for P-Erk1/Erk1, *β* = 0.030 and *η*^2^ = 0.772 for P-Erk2/Erk2, *β* < 0.001 and *η*^2^ = 0.992 for P-Akt/Akt. Brown–Forsythe test suggested the homogeneity of data variance; *P* = 0.995–0.521 for **a**, and *P* = 0.966–0.532 for **b**

We also monitored the effects of clozapine treatment on the downstream signal transducers Erk and Akt activated by their ligand stimulation (Fig. 4). In U87MG cells, the EGF-stimulated phosphorylation of Erk1, Erk2, and Akt was attenuated by clozapine in a dose-dependent manner [*F*(4, 10) = 12.49, *P* = 0.001 for Erk1; *F*(4, 10) = 7.508, *P* = 0.005 for Erk2; *F*(4, 10) = 52.81, *P* < 0.001 for Akt] (Fig. 4a). In cultured cortical neurons, the neuregulin-stimulated phosphorylation of Erk1, Erk2, and Akt was similarly inhibited by clozapine [*F*(4, 10) = 8.909, *P* = 0.002 for Erk1; *F*(4, 10) = 8.47, *P* = 0.003 for Erk2; *F*(4, 10) = 318.8, *P* < 0.001 for Akt] (Fig. 4b).

These results suggest that clozapine exerts dose-dependent negative effects not only on ErbB1/4 kinases but also on their ligand-induced downstream signaling, at least, at cellular levels. We may argue that the employed concentrations of clozapine in these culture experiments were relatively high with respect to clinical pharmacology of drug dosing^46^. We discuss the clinical rationale of whether the micromolar concentrations of clozapine are attainable in the antipsychotic medication in human patients (see Discussion).

### Direct inhibition of ErbB kinases by clozapine

**Fig. 5:**
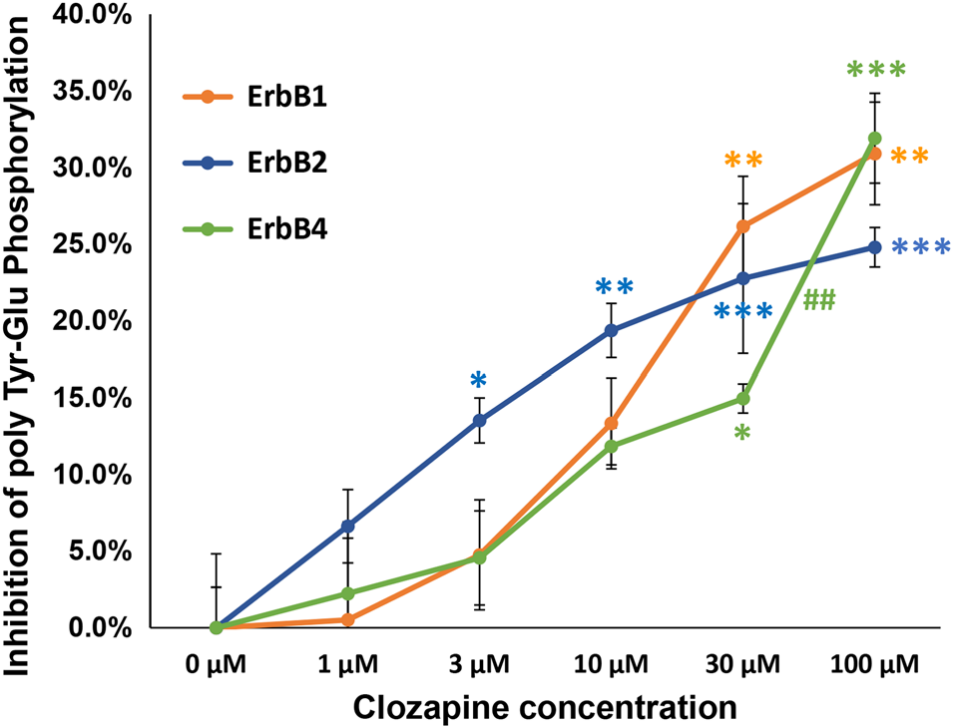
In vitro effects of clozapine on the enzyme activities of recombinant ErbB1/2/4 kinases. Recombinant intracellular domain proteins of ErbB1, B2, and B4 were obtained from the commercial sources and employed in the present in vitro kinase assay using the substrate of Tyr-Glu heteropolymers. The in vitro kinase assay was performed in the presence of 0–100 µM clozapine in the non-reducing condition (see Materials and Methods), and the product of phosphorylated tyrosine was quantitated by enzyme immunoassay with the peroxidase-conjugated anti-phosphotyrosine antibody. The graph reveals % inhibition of the ErbB kinase activity (mean ± SE, *n* = 5 assay wells), compared to the basal enzyme activity (0% inhibition). *P < 0.05, **P < 0.01, and ***P < 0.001 vs. control data (0 μM clozapine), ^##^*P* < 0.01 between adjacent concentrations, one-way ANOVA followed by Tukey’s test. *β* = 0.003 and *η*^2^ = 0.662 for ErbB1 kinase, *β* < 0.001 and *η*^2^ = 0.684 for ErbB2 kinase, *β* < 0.001 and *η*^2^ = 0.774 for ErbB4 kinase. Brown–Forsythe test suggested the homogeneity of data variance; *P* = 0.780 for ErbB1 kinase, 0.768 for ErbB2 kinase, and 0.650 for ErbB4 kinase. NRG; neuregulin-1

Several potential mechanisms may illustrate the kinase-inhibitory activity of clozapine: (1) competitive interaction with ligands for ErbB receptors, (2) inhibition of ErbB dimerization, and (3) enzymatic inhibition of ErbB kinases. Currently, *Escherichia coli*-derived recombinant proteins of truncated ErbB kinases, which lack the extracellular domains for their interaction with EGF/neuregulins, are commercially available and often utilized for cancer drug screening. Using glutamate-tyrosine heteropolymers as a substrate, the enzyme activity of the truncated ErbB1, B2, and B4 kinases was assessed in vitro in the presence of various concentrations of clozapine (Fig. 5). The tyrosine kinase activities of ErbB1 and ErbB4 were significantly decreased in the presence of 30 μM clozapine [*F*(5, 24) = 7.91, *P* = 0.001 for ErbB1 and *F*(5, 24) = 16.47, *P* < 0.001 for ErbB4]. The tyrosine kinase activity of ErbB2 was significantly reduced at 3 μM clozapine [*F*(5, 24) = 10.405, *P* < 0.001 for ErbB2].

### In vivo effects of clozapine on basal ErbB phosphorylation in rat brain

**Fig. 6:**
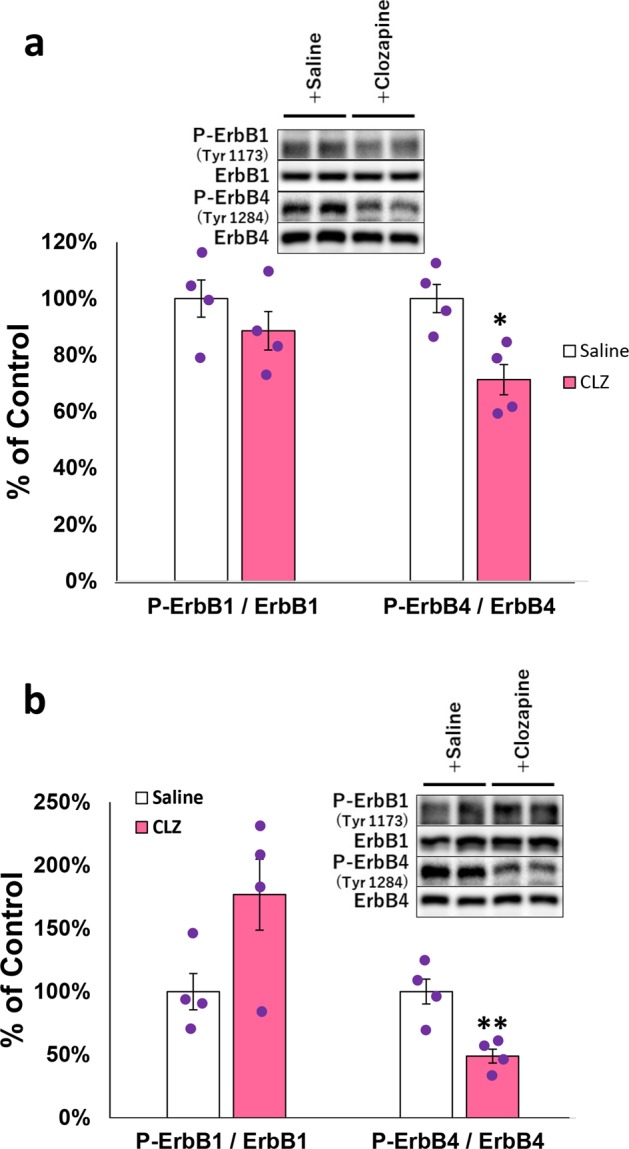
In vivo clozapine effects on basal ErbB1 and ErbB4 phosphorylation in the brain. Clozapine (20 mg/kg) or saline was intraperitoneally injected to adult rats and maintained for 1 h. Frontal cortex (**a**) and hippocampus (**b**) were taken and subjected to immunoblotting for phosphorylated Try1173 of ErbB1 (P-ErbB1) and Try1284 of ErbB4 (P-ErbB4) and for total ErbB1 and ErbB4. Typical immunoblots for phosphorylated and total ErbB1 and ErbB4 are shown as examples. The ratio of P-ErbB1 to total ErbB1 and that of P-ErbB4 to total ErbB4 in the saline-injected control rats are set to 100% (mean ± SE, *n* = 4 rats for each group). In **a** *β* = 0.856 and *η*^2^ = 0.154 for P-ErbB1/ErbB1, *β* = 0.341 and *η*^2^ = 0.808 for P-ErbB4/ErbB4. In **b** *β* = 0.575 and *η*^2^ = 0.425 for P-ErbB1/ErbB1, *β* = 0.280 and *η*^2^ = 0.902 for P-ErbB4/ErbB4. Brown–Forsythe test suggested the homogeneity of data variance; *P* = 0.936–0.603 for **a**, and *P* = 0.427–0.414 for **b**

Does clozapine exert similar actions on ErbB phosphorylation in vivo? To address this question, we acutely administered clozapine (20 mg/kg) to adult rats. The dose suitable for this animal experiment was selected from the doses that have been employed in the previous animal studies^47,48^ and reported to give a rise to a clinically relevant concentration in rat blood^49,50^ (see also Discussion). The administration of clozapine significantly reduced the basal phosphorylation levels of ErbB4 in the hippocampus and frontal cortex (Fig. 6). The effect on basal ErbB1 phosphorylation was undetectable in both brain regions in this condition, however.

## Discussion

Several antipsychotic compounds are known to exhibit the non-psychiatric or non-monoaminergic actions on various types of tissues and cells, such as the anti-carcinogenic actions^16–24^. In the present study, we attempted to elucidate these unique actions of antipsychotic compounds that cannot be illustrated by established dopaminergic and/or serotonergic antagonism. To exclude the influences of the anti-monoaminergic actions of clozapine, we employed the cultures of cancer cells, where neurotransmission is not involved. First, we compared the antiproliferative effects of four antipsychotic compounds (olanzapine, clozapine, risperidone, and haloperidol). Subsequently, we explored the molecular target(s) responsible for their antiproliferative function. In particular, we focused on the ErbB receptor tyrosine kinases since the inhibition of ErbB kinases is suggested to produce antipsychotic drug-like effects^32–34^. Among the antipsychotic compounds, clozapine gained immense attention, which possesses the unique psycopharmacological feature^4,6–8^. In agreement with our assumption, clozapine produced the most remarkable inhibition of ErbB kinase activities in this study, which might be implicated in its unique antipsychotic property and side effects.

In the present experiments, we made the following observations. (1) The growth/survival rates of U87MG and A431 cells in culture were decreased by clozapine and haloperidol. (2) The acute application of clozapine inhibited the ligand-induced phosphorylation of ErbB1 and that of ErbB4 in dose-dependent manners. (3) Clozapine also attenuated the ligand-dependent activation of Akt and Erk1/2 within its micromolar concentration ranges in culture. (5) Clozapine-dependent inhibition of ErbB1 and ErbB4 kinases was reproducible in vitro, suggesting that clozapine can directly inhibit the kinase enzymes of ErbB1 and ErbB4. (6) In vivo application of clozapine similarly downregulated the basal phosphorylation of ErbB4, but not that of ErbB1 in rat brain. Although some controversy of the clozapine action on basal ErbB phosphorylation still remains, the present results suggest that clozapine targets ErbB kinases to interfere the ligand-dependent ErbB kinase actions.

### Potential contribution of ErbB kinase inhibition to the unique antipsychotic profile of clozapine

Several postmortem studies have suggested that the hyper-signaling of ErbBs is associated with the neuropathology of schizophrenia^30,51–53^ although the etiological contributions of the up- or downregulation of ErbB signaling to schizophrenia remain to be controversial^30,31,54^. The expression of EGF, neuregulins, or ErbB receptors is reported to increase in the brain of patients with schizophrenia as well as in the blood of the patients^52,53,55–57^. Conversely, EGF concentrations in blood are decreased in patients with schizophrenia^52^. Consistent with the hypotheses, the ligands for ErbB1 and ErbB4 are known to induce various behavioral deficits in the animal models relevant to schizophrenia. Such deficits include decreased prepulse inhibition, lowed social interaction, elevated sensitivity to psychostimulants, and reduced mismatch negativity for EGF/ErbB1^57–60^ and for neuregulin-1/ErbB4^61–64^.

The inhibitors for ErbB1 kinases exert opposite functions to ameliorate the above behavioral deficits. The anti-cancer agents of ErbB inhibitors namely, ZD1839, PD153035 and OSI-774, all ameliorate the deficits of acoustic prepulse inhibition in the animal models established by neonatal EGF injection and hippocampal lesion without apparent adverse influence^32,33^. Similarly, 6-hydroxyantorathene (emodin), an attenuator of ErbB signaling, normalizes such behavioral deficits of the animal model for schizophrenia^65^. Recently, Tadmor et al., provided supportive evidence for this argument; chronic administration of an ErbB inhibitor, JNJ28871063, ameliorates the social withdrawal of phencyclidine-treated rats^34,66^.

Of note, the kinase-inhibitory activity of clozapine might also be implicated in its characteristic side effects; myocarditis and agranulocytosis. These deficits are inducible by the inhibitors for ErbB kinases as well. The attenuation of ErbB kinase signals is known to impair the normal development of cardiac myocytes and result in myocarditis in adult animals^18,67^. The anti-cancer drugs targeting ErbB kinases can produce agranulocytosis and/or neutropenia^68,69^. However, the present results do not rule out that these side effects of clozapine may involve other protein kinases or signal transducers.

Collectively, all previous reports agree with the present assumption that the unique antipsychotic action and potentially the side effects of clozapine might be associated with the observed negative action of clozapine on ErbB kinases.

### Pharmacological consideration of clozapine concentrations in the present study

The present culture results revealed that the acute application of 10–30 μM clozapine and 30 μM haloperidol significantly suppressed the ligand-triggered phosphorylation of ErbB1 and ErbB4. The in vitro kinase study revealed that the minimum effective concentration of clozapine was as low as 3–30 μM. These concentrations of clozapine in the present study raise an important question of whether the corresponding concentrations can be attained clinically in human patients with schizophrenia. According to the reported minimum effective dose of antipsychotics^70^, the concentration 30 μM of clozapine, olanzapine, haloperidol, and risperidone, used in the present cell culture experiments, are equivalent to 20, 188, 565, and 615 mg/kg of chlorpromazine, respectively. This calculation conversely suggests that the molar concentration of clozapine in cultures was the lowest and that of haloperidol was the highest with respect to conventional antipsychotic pharmacology. For example, the conventional clinical dose range of haloperidol is 0.75–6 mg/day/person, which results in the concentrations of 1–4 nM in the bloodstream of patients^71,72^. Thus, the concentration of haloperidol in the present culture experiments was markedly higher than the clinical dose used for patients with schizophrenia. Thus, a regular clinical use of haloperidol is unlikely to influence ErbB signaling in patients.

In contrast, the accepted daily clinical dose of clozapine (i.e., 50–600 mg/day) is much higher than that of haloperidol. In contrast to haloperidol, the effective concentration of clozapine used in the present culture study, 10–30 μM, appears to be clinically acceptable and/or attainable. The conventional dose range of clozapine in patients’ blood is around 0.3–7.8 µM^73^. Using adult rats, Baldessarini et al.^50^ reported that single intraperitoneal injection of clozapine (20 mg/kg, same as the present dose) to rats resulted in 2.2 μM concentration in blood and 47 μM concentration in brain, which is higher than the effective dose (i.e., 30 μM) obtained in the present culture experiments. Moreover, chronic administration of clozapine is reported to further elevate its local concentrations in the brain owing to its lipophilic accumulation^50,74,75^. Thus, we speculate that the clinical use of clozapine, in particular, in case of high dosing and chronic administration, would allow it to reach the micromolar concentrations and affect ErbB signaling in human brain.

### The controversial actions of clozapine on basal ErbB1 phosphorylation and its downstream signaling

In the present experiments, the ligand-dependency of the clozapine actions differed between its target molecules of ErbB1 and ErbB4 kinases. In culture as well as in vivo, clozapine inhibited both the neuregulin-triggered and untriggered (basal) phosphorylation of ErbB4. In contrast, clozapine failed to affect the basal phosphorylation of ErbB1. Thus, clozapine appears to be ineffective on the ligand-independent basal phosphorylation of ErbB1.

Is this phenomenon discrepant against the result from the in vitro kinase assay of ErbB1 kinase? The ligand-induced phosphorylation of ErbB receptors mainly depend on the enzyme activity of their receptor kinases. However, it is not necessary that the basal phosphorylation of ErbB receptors should depend on their homodimerization and trans-phosphorylation. It is because several other tyrosine kinases such as Src, Met, or PDGFR are known to induce ErbB1 phosphorylation through their heteromeric dimerization or binding^31^. The above controversy in clozapine actions on basal phosphorylation of ErbB1 might be ascribed to clozapine-insensitive tyrosine kinases, other than ErbB kinases.

We also attempted to illustrate the paradoxical positive and negative actions of clozapine on ErbB phosphorylation and signaling^18,22,27^. When ErbB1 was stimulated by its full ligand EGF, clozapine works as an inhibitor for the kinase in the present study, while clozapine itself is reported to stimulate ErbB1 kinase or Erk/Akt signaling in vivo^26,28,49^. Smith et al.^27^ proposed that the clozapine-triggered activation of Akt signaling involves insulin secretion in vivo while Pereira et al.^22^ suggest that the clozapine-triggered activation of Erk signaling in vivo is mediated by ErbB1 (EGFR) transactivation. In the brain in vivo, where robust strengths of neurotransmission occur, the direct and indirect effects on ErbB signaling and monoaminergic neurotransmission would lead to such complex and controversial phenomena. Even in the present experiments, the controversial results of the clozapine actions were obtained; in cultured A431 cells, 100 μM clozapine reduced EGF-trigged ErbB1 phosphorylation to 66.2 ± 8.3% (Supplementary Fig. S4) whereas the same compound elevated basal ErbB1 phosphorylation by twofold in the absence of EGF (Supplementary Fig. S5). Therefore, clozapine appears to behave like an inverse agonist for ErbB1 kinase, although the appearance of the partial agonistic effects of clozapine on ErbB1 depends on the target cell types (not seen in U87MG cells; data not shown).

The agonistic action of clozapine on ErbB kinases is in agreement with the previous argument that the activation of Akt signaling in the brain is beneficial for antipsychotic medication^76^. However, as the expression of ErbB kinases is relatively limited to the subpopulation of brain cells, it would be difficult for the agonistic action of clozapine on ErbB receptors to fully illustrate the reported global effects of clozapine on Akt signaling in the brain^25–27^. The molecular mechanism underlying these bi-directional actions of clozapine on ErbB kinases remains to be characterized.

In summary, the present study proposes the possibility that clozapine has a novel biological reactivity with ErbB1 and ErbB4 kinases, which might be associated with the unique psychopharmacological profiles of this drug. With the reported unique chemical reactivity of clozapine^77^, future studies hopefully warrant molecular understanding of the interaction between clozapine and ErbB kinases.

## Supplementary information

Supplemental Figure S1- S6
