# Supplementary material for: Clozapine-dependent inhibition of EGF/neuregulin receptor (ErbB) kinases
Source: Transl Psychiatry. 2019 Aug 1;9:181. doi: 10.1038/s41398-019-0519-1 (PMC6675791; doi:10.1038/s41398-019-0519-1)
Supplement: Supplementary file 1 — Supplemental Figure S1- S6 [file 41398_2019_519_MOESM1_ESM.pdf]

Supplemental Materials;
Supplemental Figures

Clozapine-dependent inhibition of EGF/neuregulin receptor (ErbB) kinases

Yutaro Kobayashi, Yuriko Iwakura, Hidekazu Sotoyama, Eiko Kitayama, Nobuyuki Takei, Toshiyuki Someya, Hiroyuki Nawa

Page 2; **Figure S1**. Dose-dependent effects of clozapine on the growth/survival of cancer cells.

Page 3; ; **Figure S2.** The antipsychotic alone effects on basal ErbB phosphorylation by in cultured cortical neurons.

Page 4-5; **Figure S3. E**ffects of clozapine on phosphorylation inhibition of ErbB tyrosine residues.
.
Page 6; **Figure S4.** Effects of clozapine doses on phosphorylation levels of ErbB1, Erk1/2, and Akt in A431 cells.

Page 7; **Figure S5.** The induction of ErbB1 phosphorylation by clozapine alone.

Page 8-30**; Figure S6.** Original immunoblot images used for figure display and statistics

Figure S1

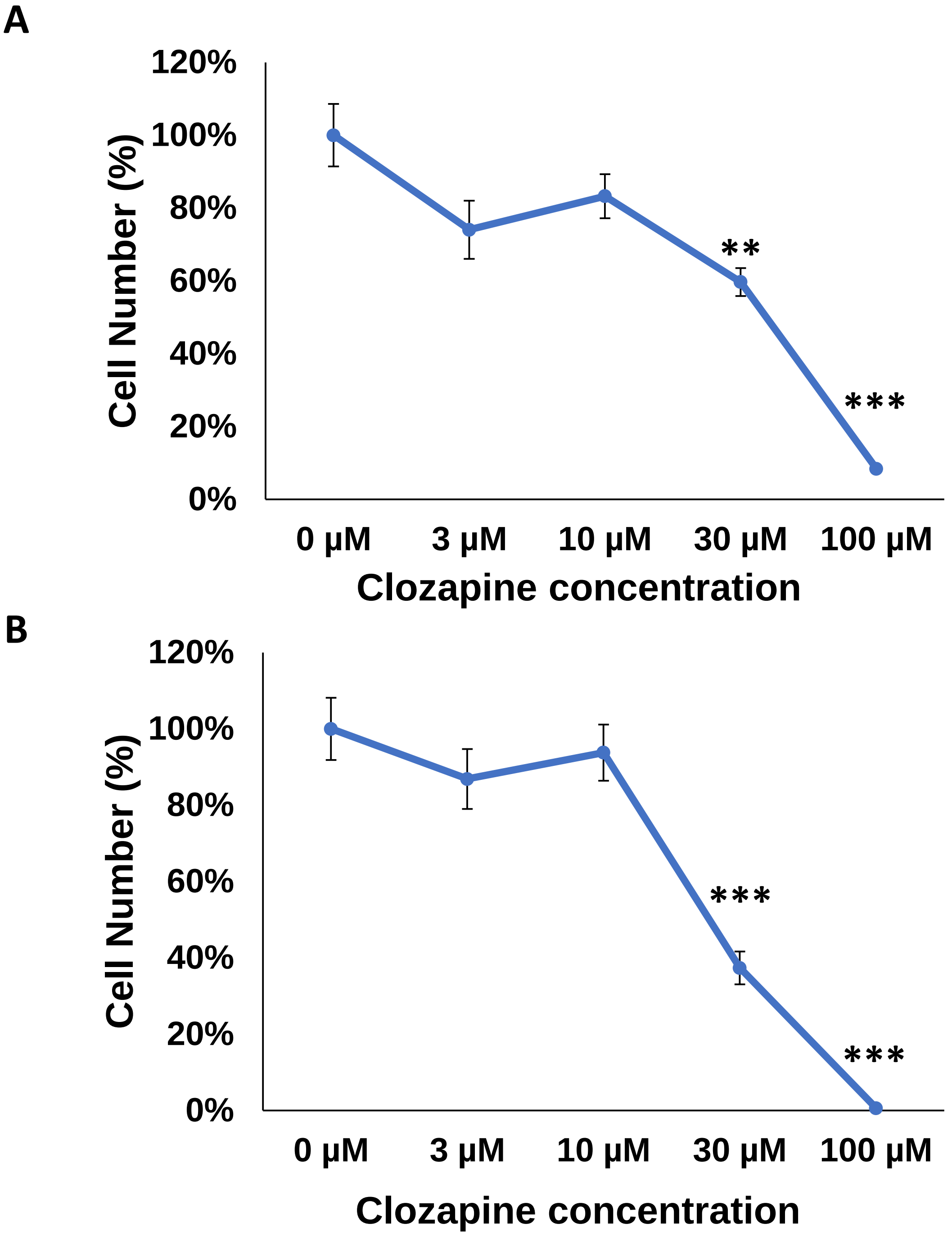

Figure S1. Dose-dependent effects of clozapine on the growth/survival of cancer cells. U87MG (A) and A431 (B) cells were grown in serum containing medium in the presence of clozapine (0 – 100 μM) for 48 h. The effects of clozapine on their cell growth/survival were estimated by a cell counting kit (CCK-8). The graph reveals % ratio of the number of living cells to that of the control culture (0 μM clozapine; 100% maximum) (mean ± SEM, n = 6 cultures). , Brown-Forsythe test suggested the homogeniety of variance among groups for A (P = 0.073) but not for B (P = 0.006). In A, **P < 0.01 and ***P < 0.001 vs DMSO, one-way ANOVA followed by Tukey's test with $\beta < 0.001$ and $\eta^2 = 0.812$. In B, Brown-Forsythe test suggested the difference in data variance among groups (P = 0.006) and thus we did not employ ANOVA. *P < 0.05/4, **P < 0.01/4 and ***P < 0.001/4 vs DMSO, by repeated Welch t-test with Bonferroni's compensation; $\beta = 0.000 – 0.920$ and $d = –0.326 – –7.009$

Figure S2

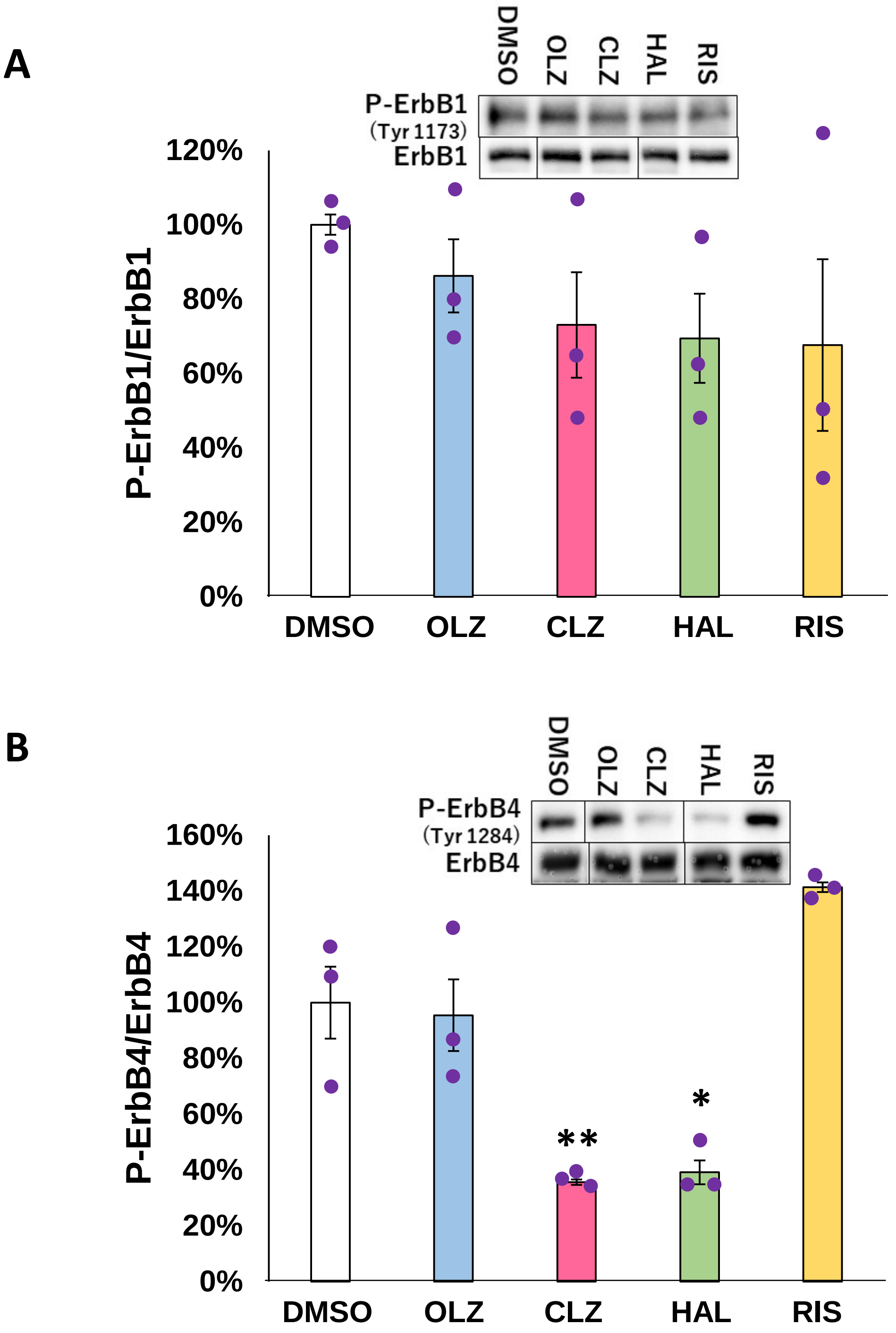

Figure S2. The antipsychotic alone effects on basal ErbB phosphorylation by in cultured cortical neurons. Cultured cortical neurons were exposed to 100 µM antipsychotics without EGF or neuregulin. Effects on ErbB1 phosphorylation (A) and ErbB4 phosphorylation (B) were measured by immunoblotting. The bar represents % ratio of the ErbB1 or ErbB4 phosphorylation levels to their basal levels (mean ± SEM, n = 3 sister cultures). $*p < 0.05$, and $**p < 0.01$, one-way ANOVA followed by Tukey's test. Brown-Forsythe test suggested the homogeneity of data variance; P = 0.681 for A and 0.456 for B. $\beta = 0.852$ and $\eta^2 = 0.203$ for A, $\beta < 0.001$ and $\eta^2 = 0.883$ for B

Figure S3-1

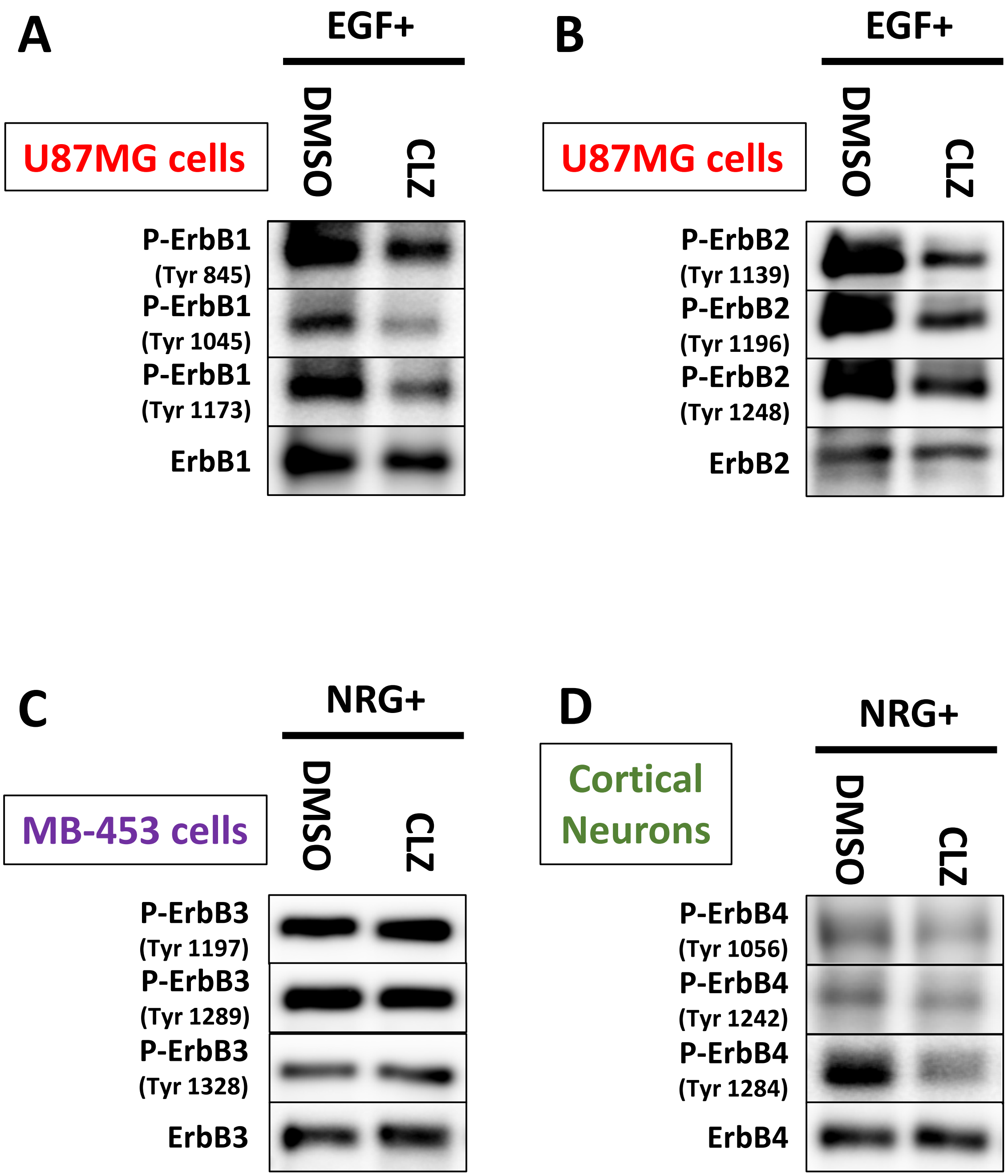

A
EGF+
U87MG cells
DMSO
CLZ
P-ErbB1 (Tyr 845)
P-ErbB1 (Tyr 1045)
P-ErbB1 (Tyr 1173)
ErbB1
B
EGF+
U87MG cells
DMSO
CLZ
P-ErbB2 (Tyr 1139)
P-ErbB2 (Tyr 1196)
P-ErbB2 (Tyr 1248)
ErbB2
C
NRG+
MB-453 cells
DMSO
CLZ
P-ErbB3 (Tyr 1197)
P-ErbB3 (Tyr 1289)
P-ErbB3 (Tyr 1328)
ErbB3
D
NRG+
Cortical Neurons
DMSO
CLZ
P-ErbB4 (Tyr 1056)
P-ErbB4 (Tyr 1242)
P-ErbB4 (Tyr 1284)
ErbB4

Figure S3-2

E

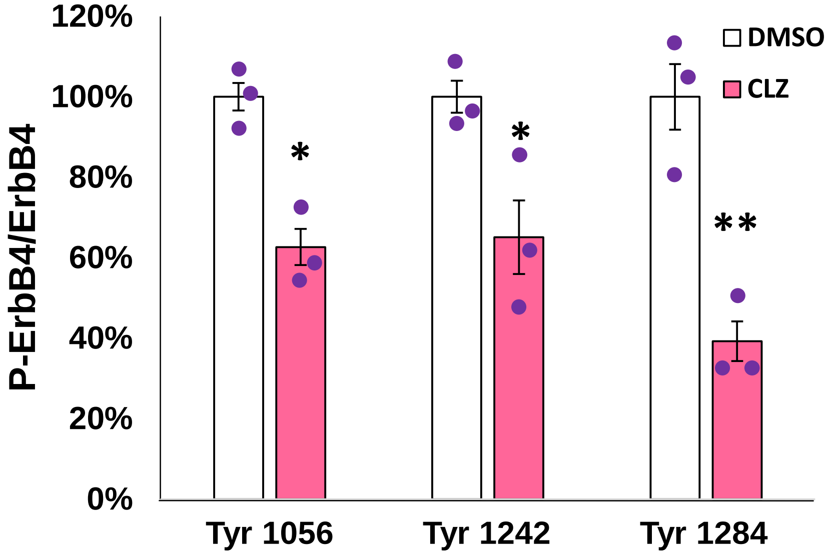

Figure S3 Effects of clozapine on phosphorylation inhibition of ErbB tyrosine residues. (A) Effects of 30 μM clozapine on EGF-triggered phosphorylation of various ErbB1 tyrosine residues were determined in U87MG cells. Phosphorylation levels of Tyr845, Tyr1045, and Tyr1173 of ErbB1 are depicted. (B) Effects of 30 μM clozapine on EGF-triggered phosphorylation of various ErbB2 tyrosine residues were examined in U87MG cells. Phosphorylation levels of Tyr1139, Tyr1196, and Tyr1248 of ErbB2 are depicted. (C) Effects of 30 μM clozapine on neuregulin-triggered phosphorylation of various ErbB3 tyrosine residues were examined in MDA-MB-453 cells. Phosphorylation levels of Tyr1197, Tyr1289, and Tyr 1328 of ErbB3 are depicted. (D) Effects of clozapine on ErbB4 tyrosine phosphorylation are compared among its tyrosine residues. ErbB4 phosphorylation was triggered by neuregulin-1 application to cultured cortical neurons. Phosphorylation levels of Tyr1056, Tyr 1242, and Tyr1284 of ErbB4 are depicted. (E) The bar in the graph represents the ratio to the neuregulin-1-induced ErbB4 phosphorylation levels (mean ± SEM, n = 3 cultures). *$P < 0.05/3$ and ***$P < 0.001/3$, two-way ANOVA followed by paired Bonferroni's test. There is no significant interaction between ErbB4 tyrosine residues and clozapine treatment.
NRG; neuregulin-1 , SEM; standard error. Brown-Forsythe test suggested the homogeneity of data variance in E; $P = 0.838$. $\beta = 0.001$ and $\eta^2 = 0.835$ for E

Figure S4

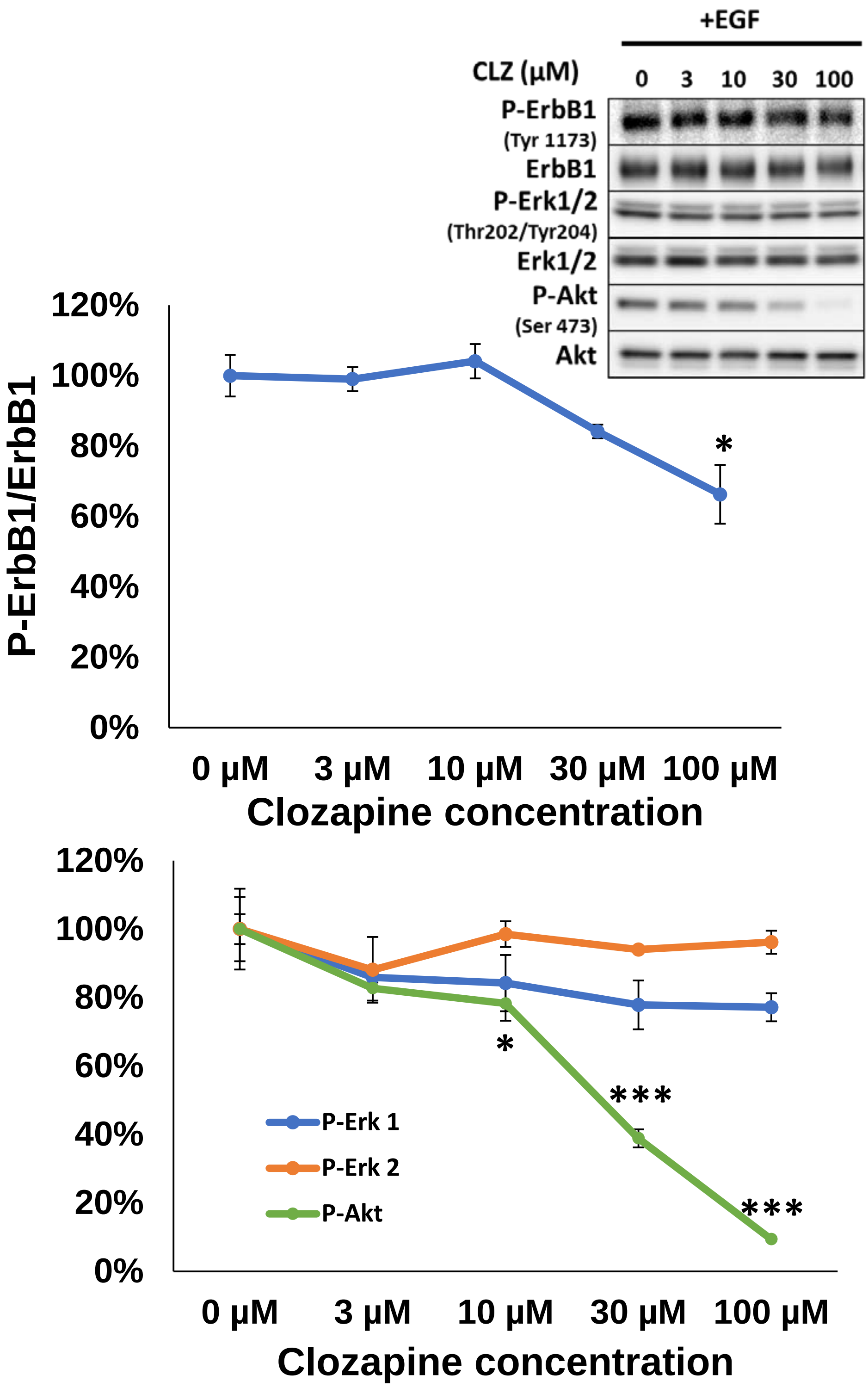

Figure S4. Effects of clozapine doses on phosphorylation levels of ErbB1, Erk1/2, and Akt in A431 cells.A431 cells were exposed to EGF in the presence of 0 – 100 μM clozapine and the phosphorylation levels of ErbB1, Erk1/2, and Akt were determined and standardized with total amounts of ErbB1, Erk1/2, and Akt. Solid lines represent % ratio of their phosphorylation levels to the maximum phosphorylation level (100%) induced by EGF alone (mean ± SEM, n = 3 cultures). *P < 0.05, and ***P < 0.001, one-way ANOVA followed by Tukey's test. In A, $\beta = 0.13$ and $\eta^2 = 0.694$ for P-ErbB1/ErbB1, and in B, $\beta = 0.737$ and $\eta^2 = 0.334$ for P-Erk1/Erk1, $\beta = 0.910$ and $\eta^2 = 0.101$ for P-Erk2/Erk2, $\beta < 0.001$ and $\eta^2 = 0.965$ for P-Akt/Akt. Brown-Forsythe test suggested the homogeneity of data variance; P = 0.784 for P-ErbB1/ErbB1, 0.686 for P-Erk1/Erk1, 0.287 for P-Erk2/Erk2, and 0.698 for P-Akt/Akt.

Figure S5

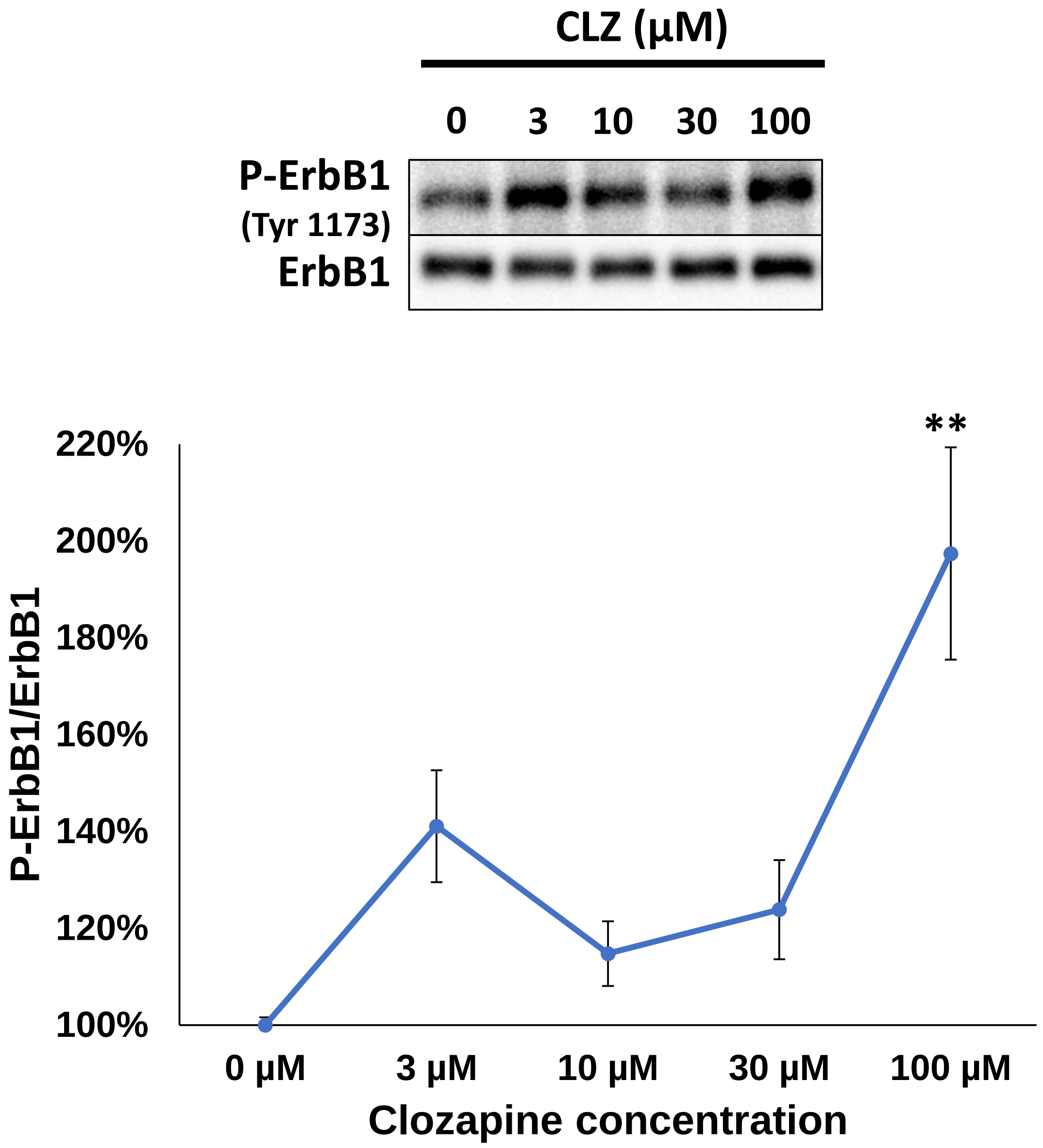

Figure S5. The induction of ErbB1 phosphorylation by clozapine alone in A431 cells. A431 cells in culture were exposed only to 0 – 100 μM clozapine and the effects on ErbB1 phosphorylation were measured by immunoblotting. A solid line represents % ratio of ErbB1 phosphorylation levels to the maximum basal levels (100%) (mean ± SEM, n = 3 cultures). **P < 0.01, one-way ANOVA followed by Tukey's test. Brown-Forsythe test suggested the homogeneity of data variance (P = 0.754). $\beta = 0.100$ and $\eta^2 = 0.712$

- **Figure S6. Original immunoblot images used for figure display and statistics.**

- Chemiluminescence images were taken at various time durations by a 16-bit CCD camera system, G:Box Chemi XRQ (Syngene, Cambridge, UK). The intensity of the immunoreactive band was normalized to the background signal surrounding the corresponding area and automatically quantitated with an image analysis software, GeneTools (Syngene). The signal intensity of multiple membranes was compensated among blots with that of an internal standard (marked as “Standard” in pictures) and compared with that of the positive controls stimulated only with the ligands for ErbB1/4. The original chemiluminescence images are presented with the automatic tone compensation of the GeneTools software. The regions of the immunoblots used for the main figures and/or statistical calculation are marked with red and blue windows, respectively. 30 µM olanzapine (OLZ), 30 µM clozapine (CLZ), 30 µM haloperidol (HAL), 30 µM risperidone (RIS), or 0.1% DMSO (vehicle for clozapine) was added prior to the stimulation of EGF or neuregulin.

Figure S6-1

A

B

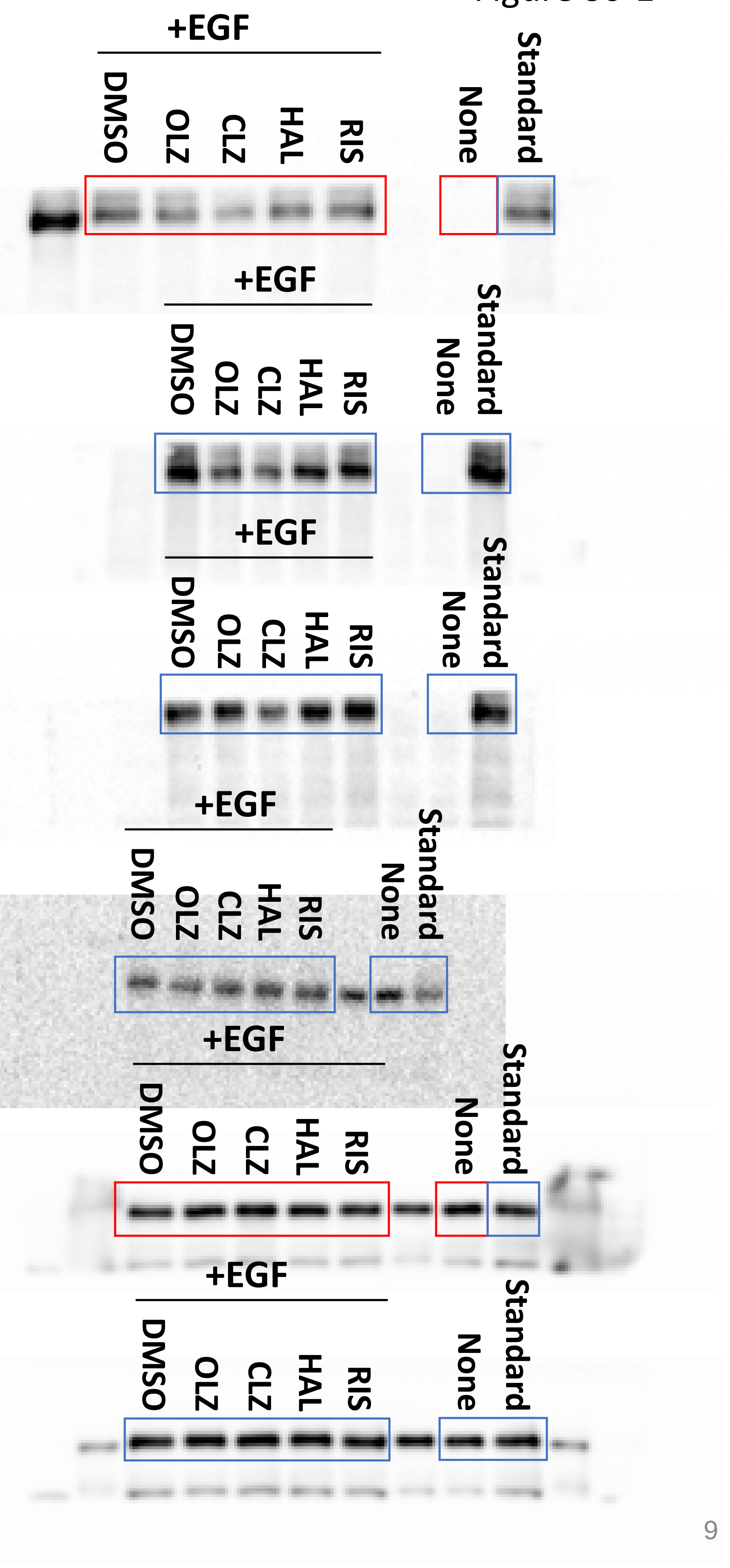

Figure S6A, S6B. Original western blot images of P-ErbB1 (A) or ErbB1 (B) used in Figure 2a. The parts surrounded by the red frames were used for the calculation and the display. The areas surrounded by the blue frames were used for the calculation.

Figure S6-2

C

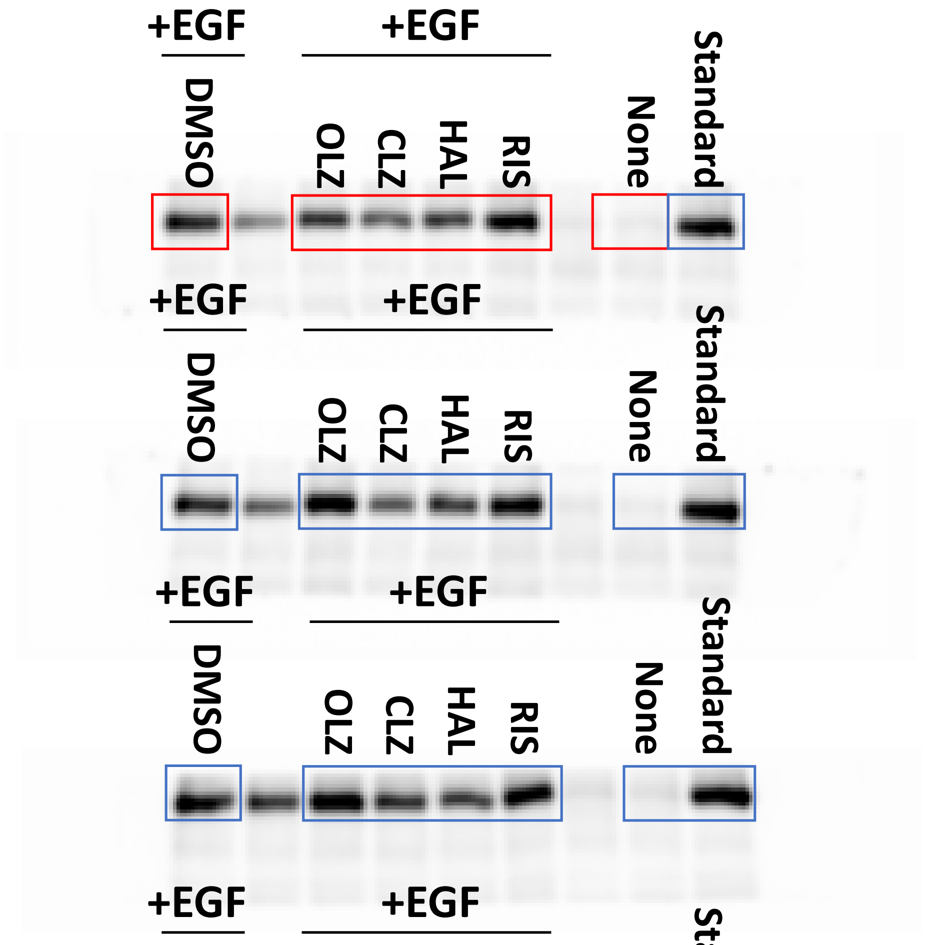

D

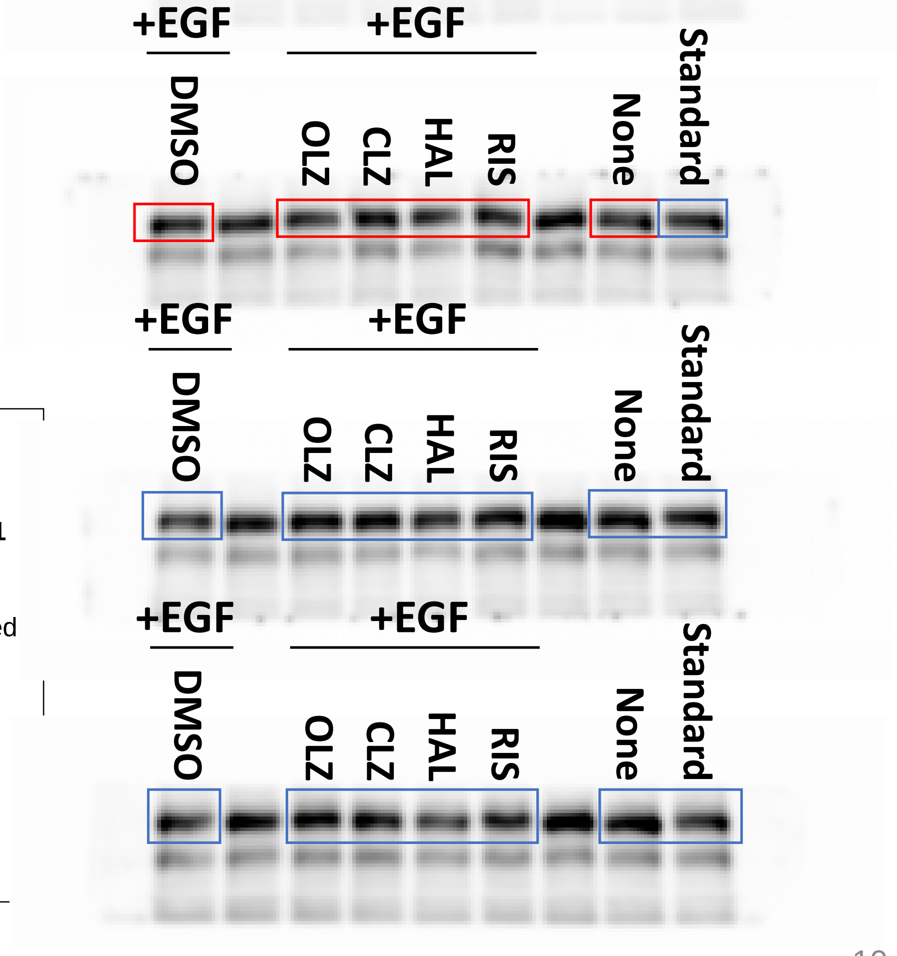

Figure S6C, S6D. Original western blot images for P-ErbB1 (C) or ErbB1 (D) used in Figure 2b.
The parts surrounded by the red frames were used for the calculation and the display. The parts surrounded by the blue frames were used for the calculation.

E

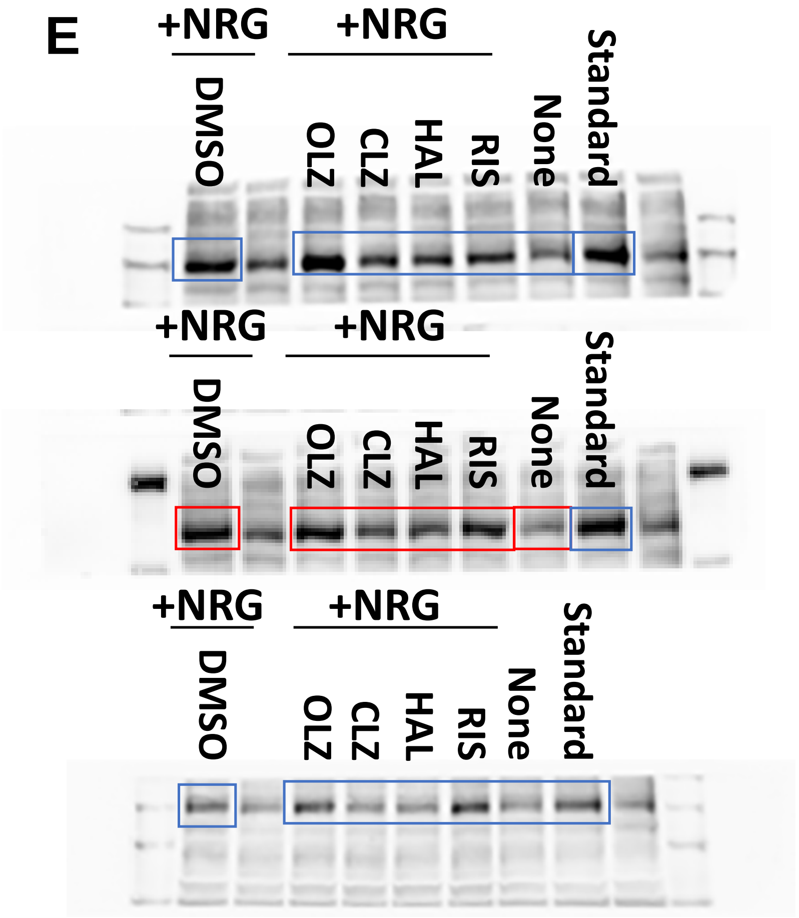

F

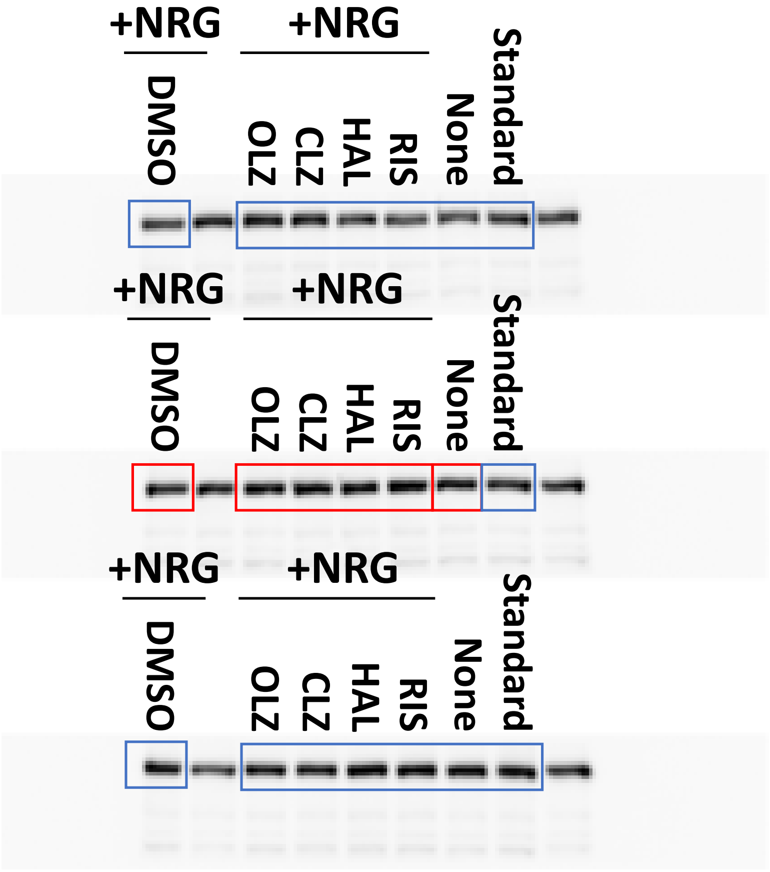

Figure S6E, S6F. Original western blot images for P-ErbB4 (E) or ErbB4 (F) used in in Figure 2c.
The parts surrounded by the red frames were used for the calculation and the display. The parts surrounded by the blue frames were used for the calculation.

Figure S6-4

G

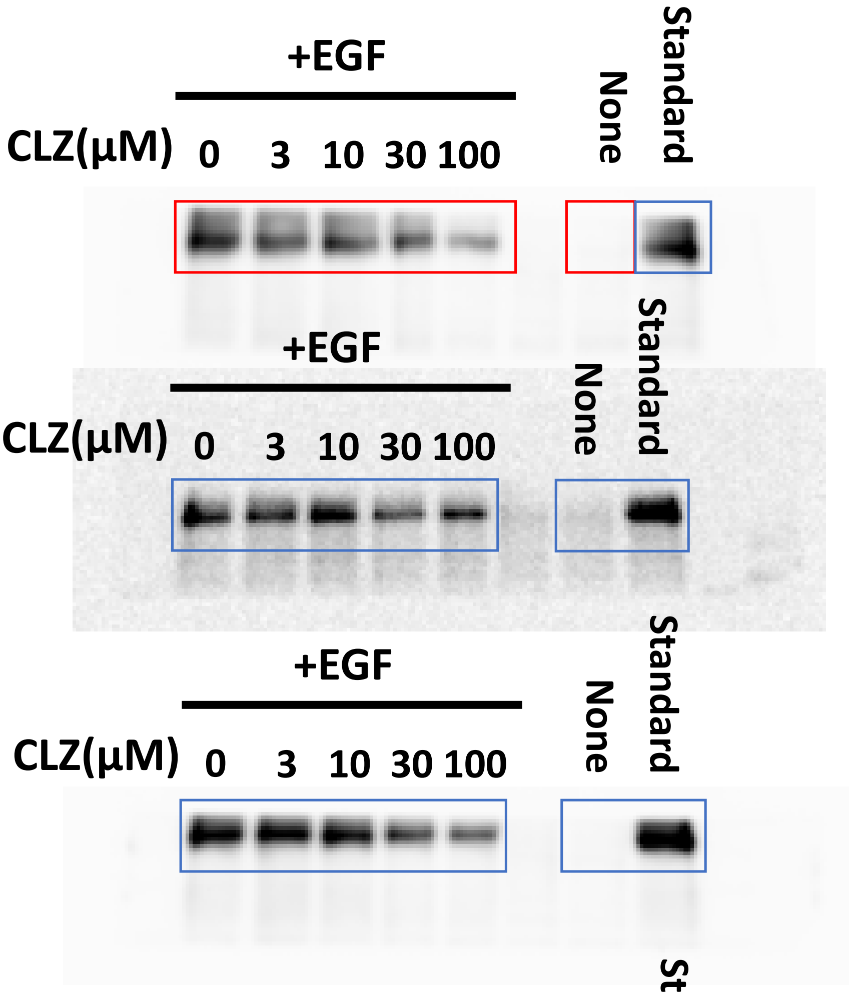

H

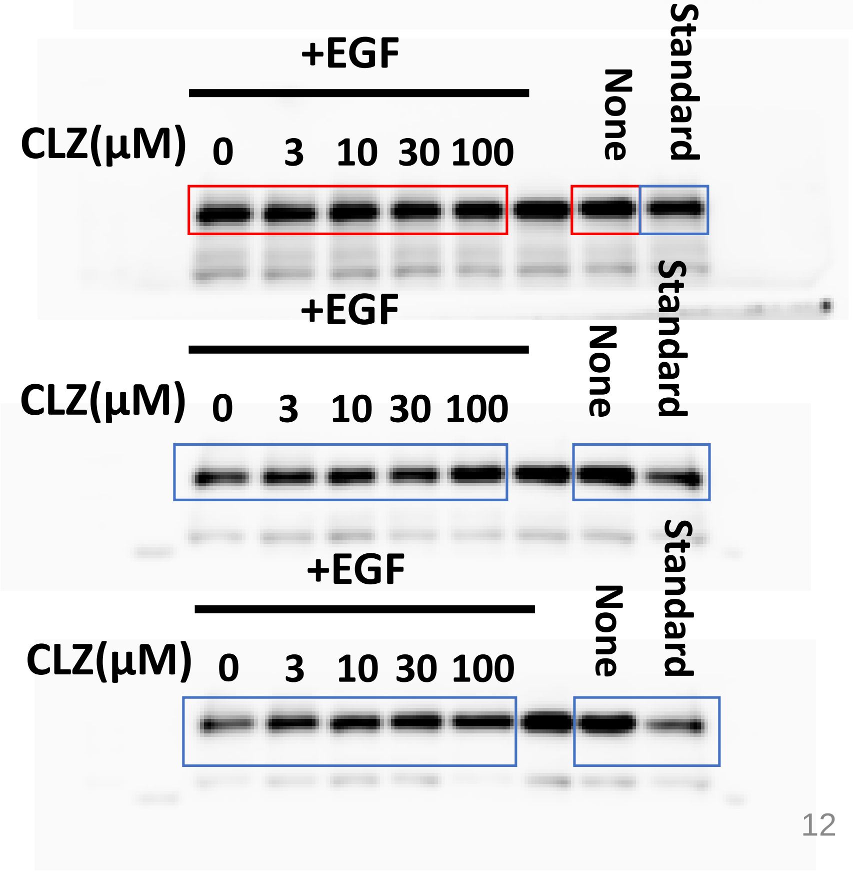

Figure S6G, S6H. Original western blot images for P-ErbB1 (G) or ErbB1 (H) used in Figure 3a. The parts surrounded by the red frames were used for the calculation and the display. The parts surrounded by the blue frames were used for the calculation.

Figure S6-5

I

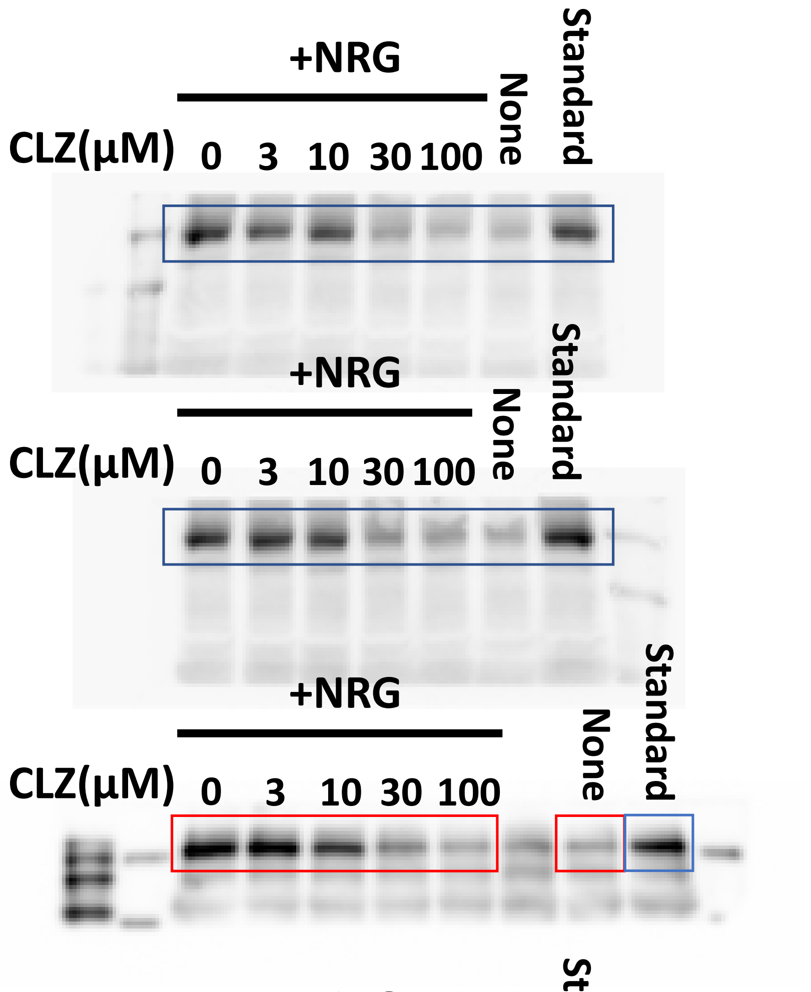

J

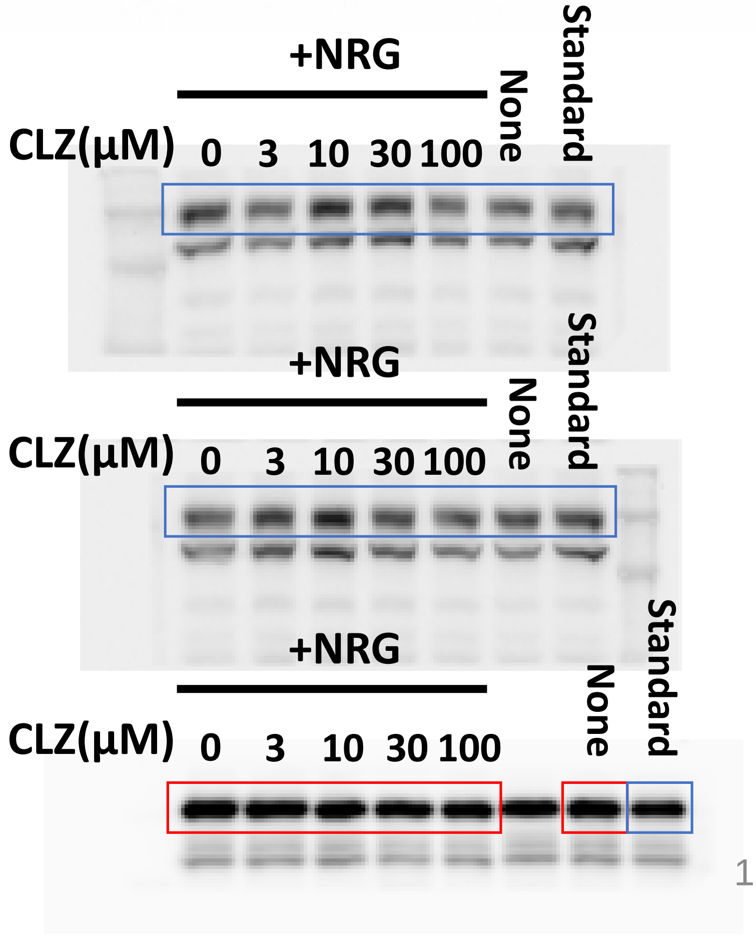

Figure S6I, S6J. Original western blot images for P-ErbB4 (I) or ErbB4 (J) used in Figure 3b. The parts surrounded by the red frames were used for the calculation and the display. The parts surrounded by the blue frames were used for the calculation.

Figure S6-6

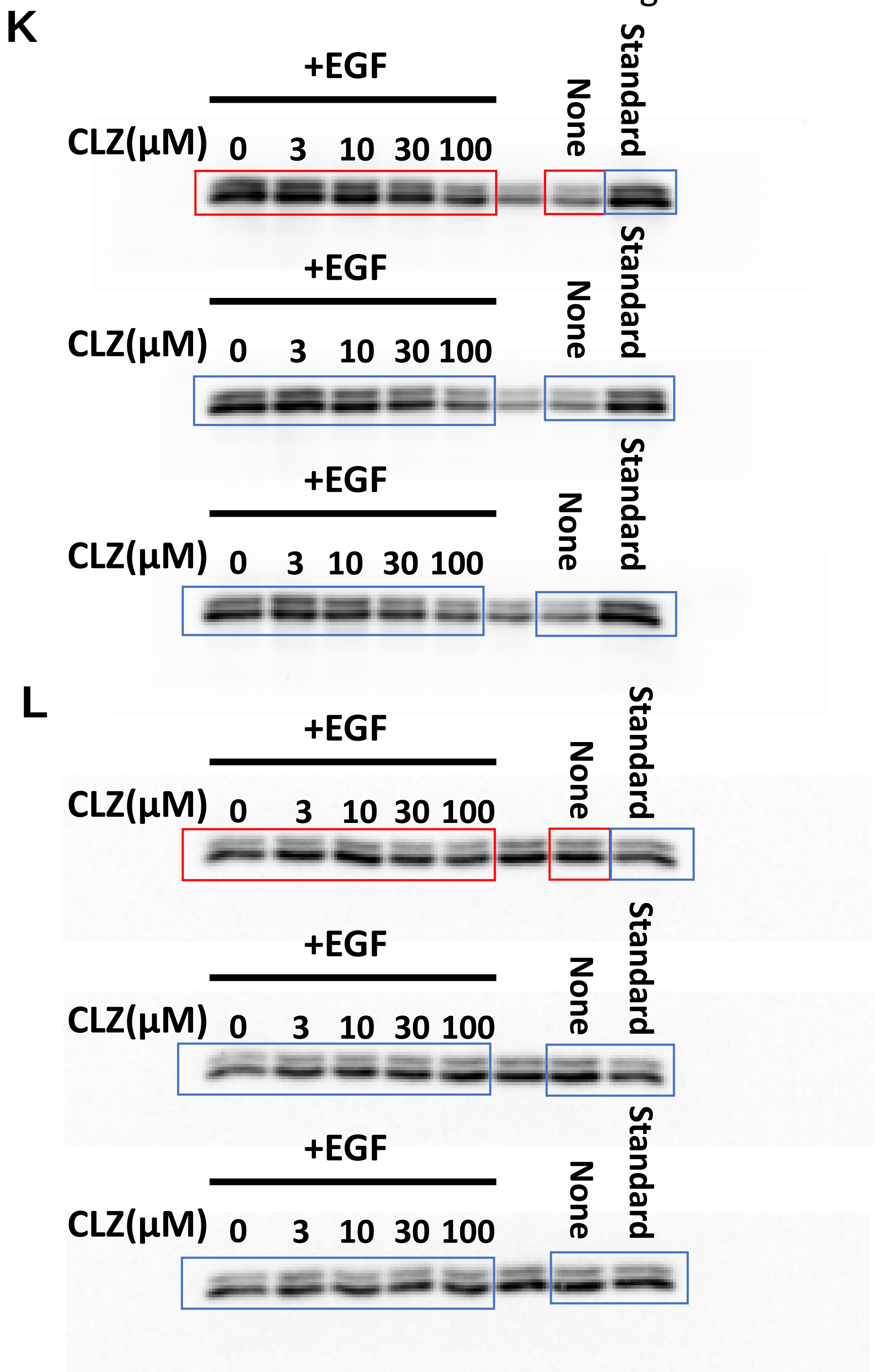

Figure S6K, S6L. Original western blot images for P-Erk (K) or Erk (L) used in Figure 4a. The parts surrounded by the red frames were used for the calculation and the display. The parts surrounded by the blue frames were used for the calculation.

Figure S6-7

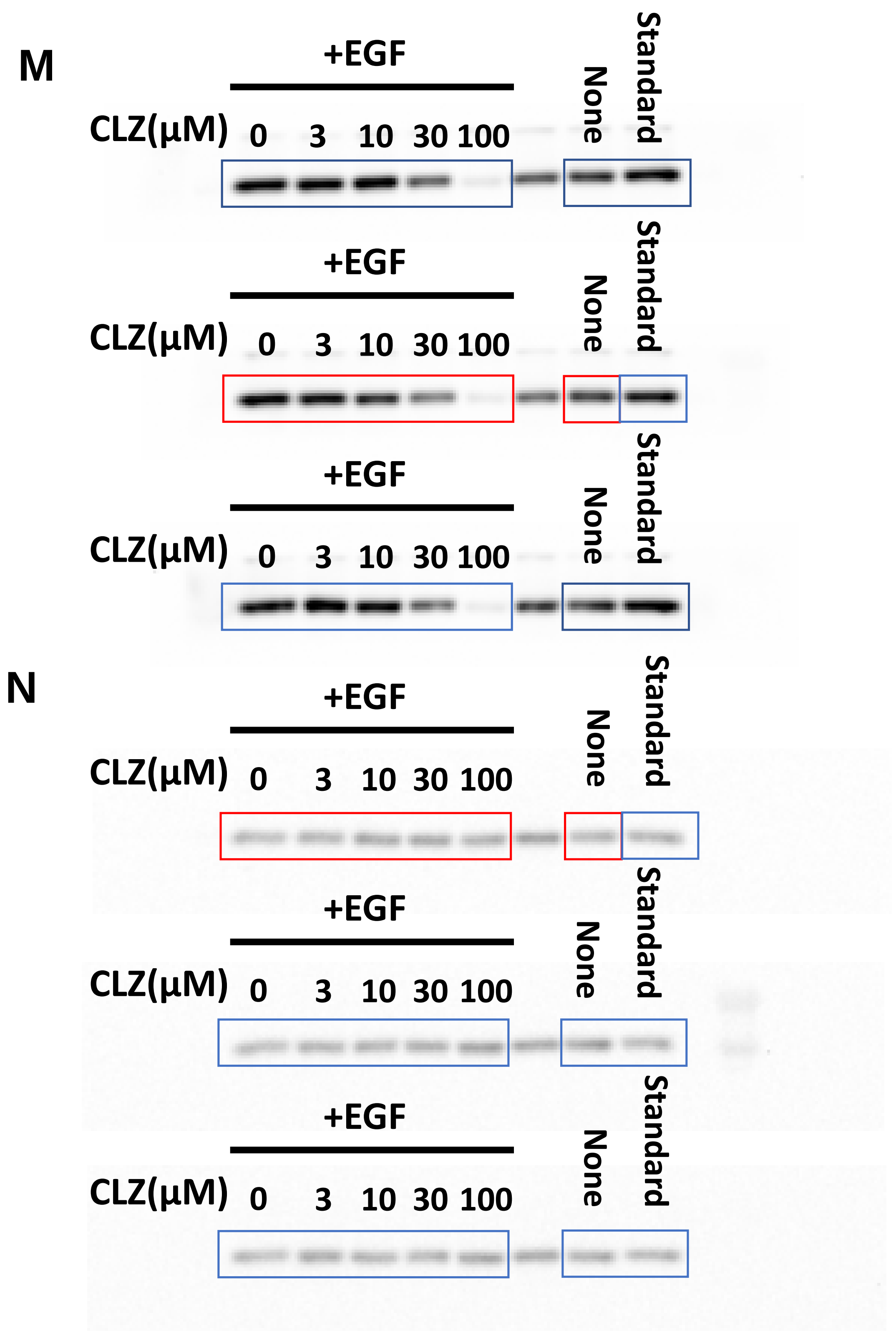

Figure S6M, S6N. Original western blot images for P-Akt (M) or Akt (N) used in Figure 4a. The parts surrounded by the red frames were used for the calculation and the display. The parts surrounded by the blue frames were used for the calculation**.**

Figure S6-8

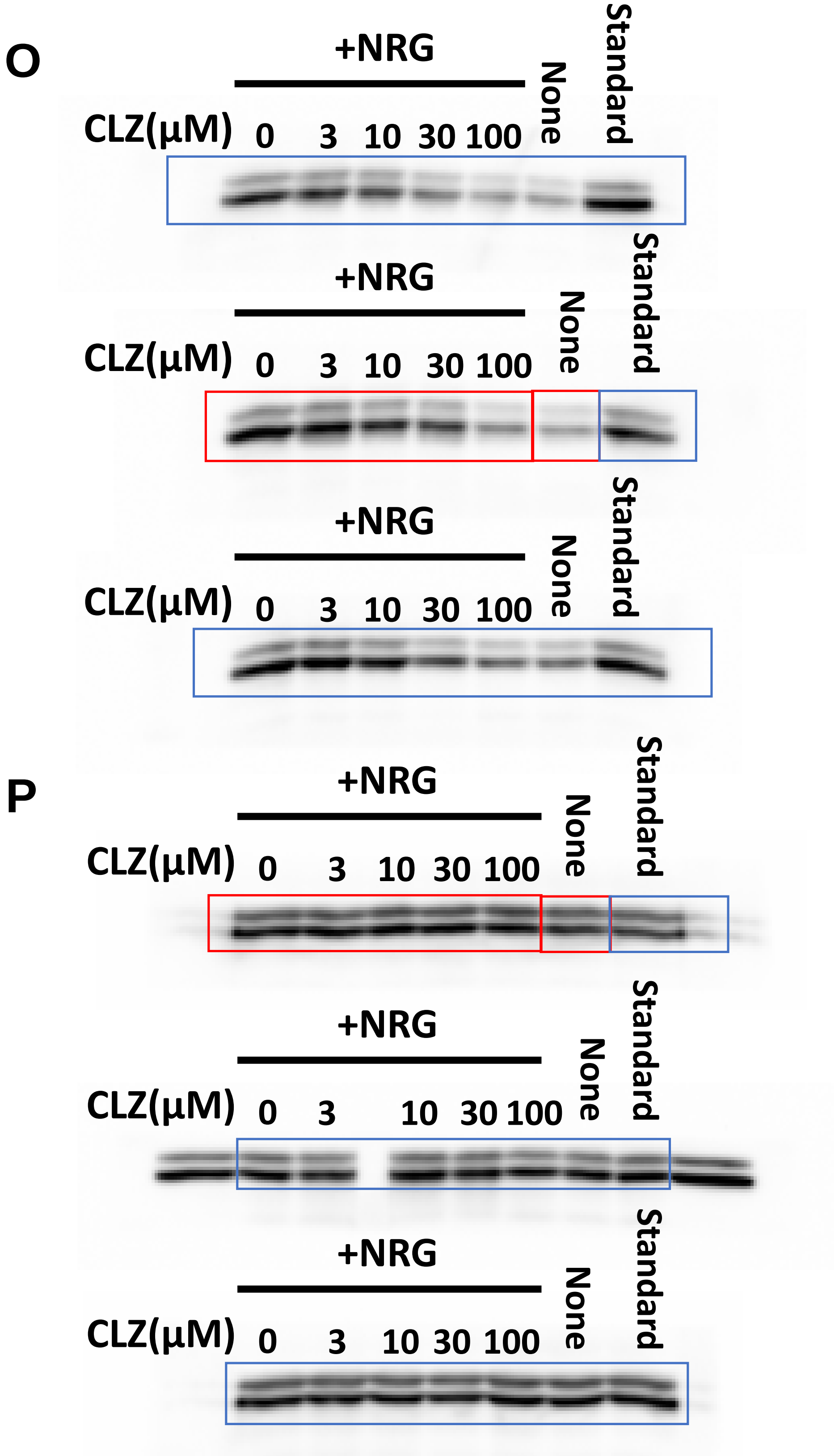

Figure S6O, S6P. Original western blot images for P-Erk (O) or Erk (P) used in Figure 4b. The parts surrounded by the red frames were used for the calculation and the display. The parts surrounded by the blue frames were used for the calculation.

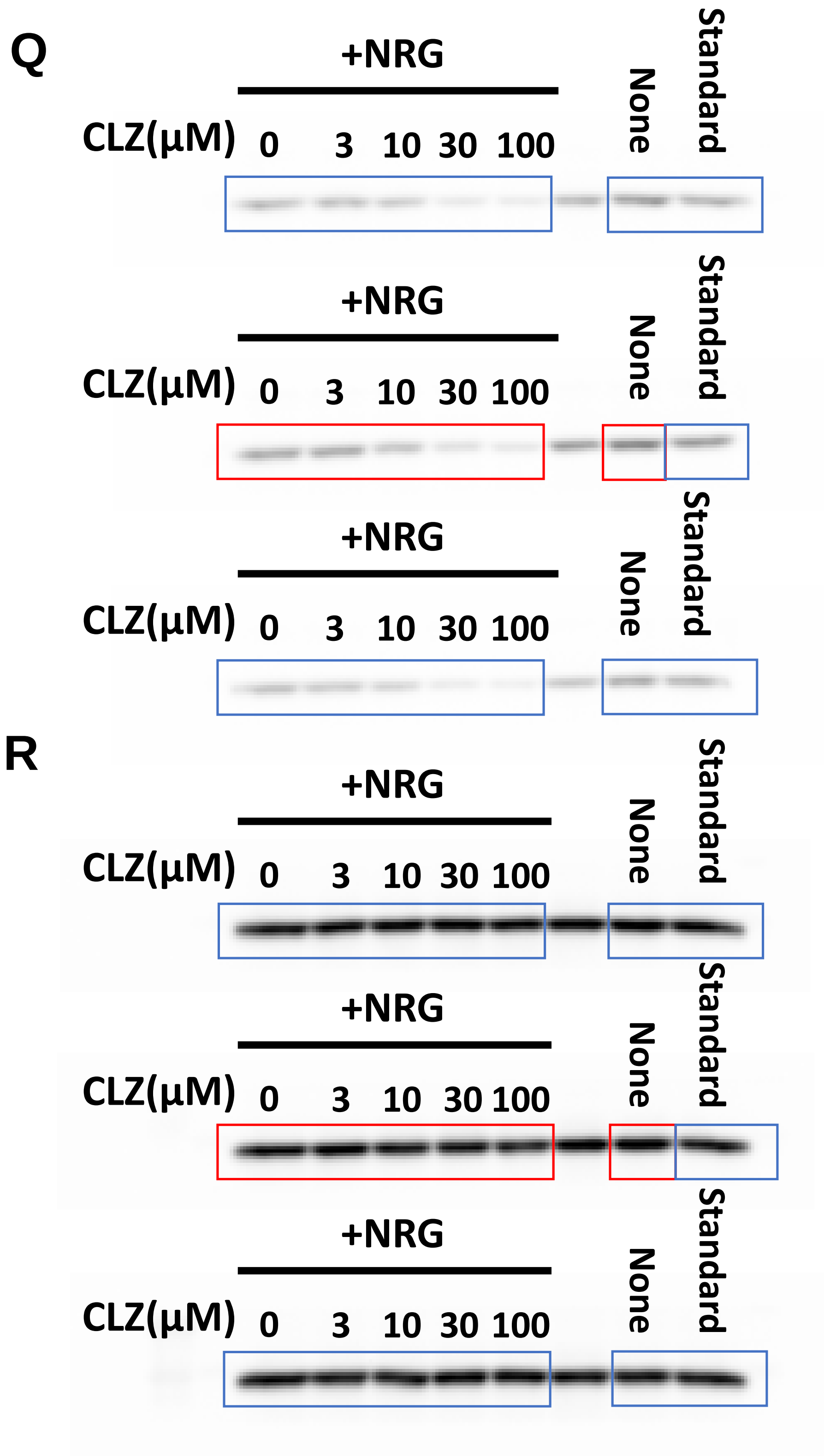

Figure S6Q, S6R. Original western blot images for P-Akt (Q) or Akt (R) used in Figure 4b. The parts surrounded by the red frames were used for the calculation and the display. The parts surrounded by the blue frames were used for the calculation.

Figure S6-10

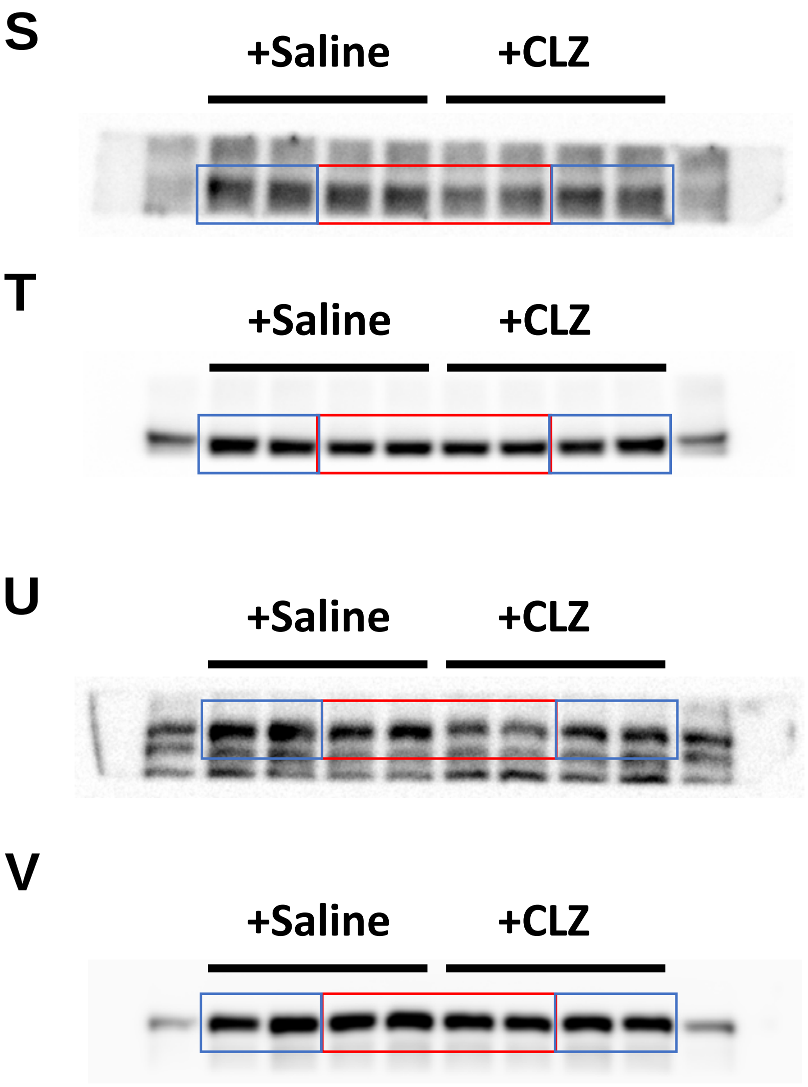

Figure S6S-S6V. Original western blot images for P-ErbB1 (S), ErbB1 (T), P-ErbB4 (U), or ErbB4 (V) used in Figure 6a. The parts surrounded by the red frames were used for the calculation and the display. The parts surrounded by the blue frames were used for the calculation. The values of the phosphorylated molecules were divided by the values of the non-phosphorylated molecules.

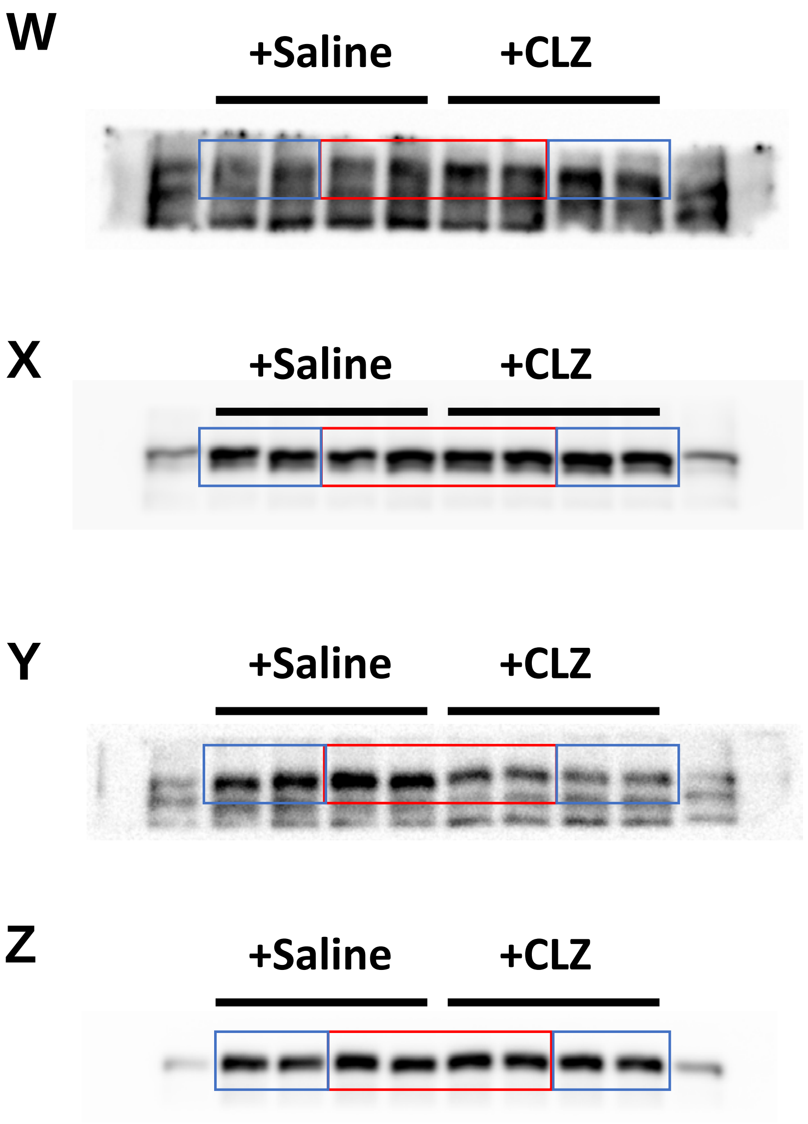

Figure S6W-S6Z. Original western blot images for P-ErbB1 (W), ErbB1 (X), P-ErbB4 (Y), or ErbB4 (Z) used in Figure 6b. The parts surrounded by the red frames were used for the calculation and the display. The parts surrounded by the blue frames were used for the calculation. The values of the phosphorylated molecules were divided by the values of the non-phosphorylated molecules.

AA

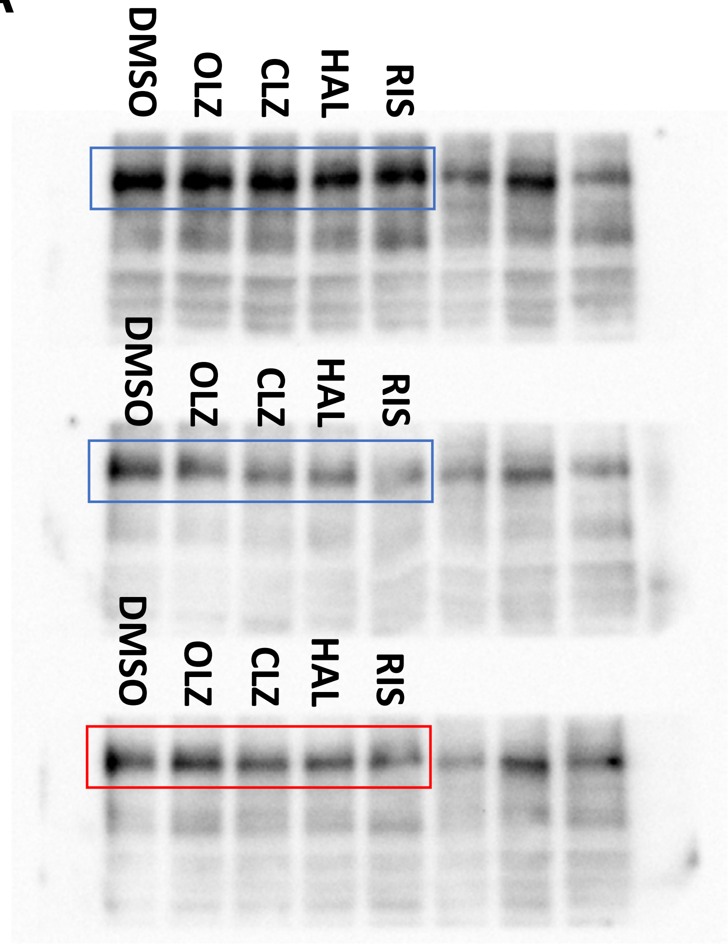

Figure S6AA. Original western blot images for P-ErbB1 (AA) used in Figure S2A. The parts surrounded by the red frames were used for the calculation and the display. The parts surrounded by the blue frames were used for the calculation.

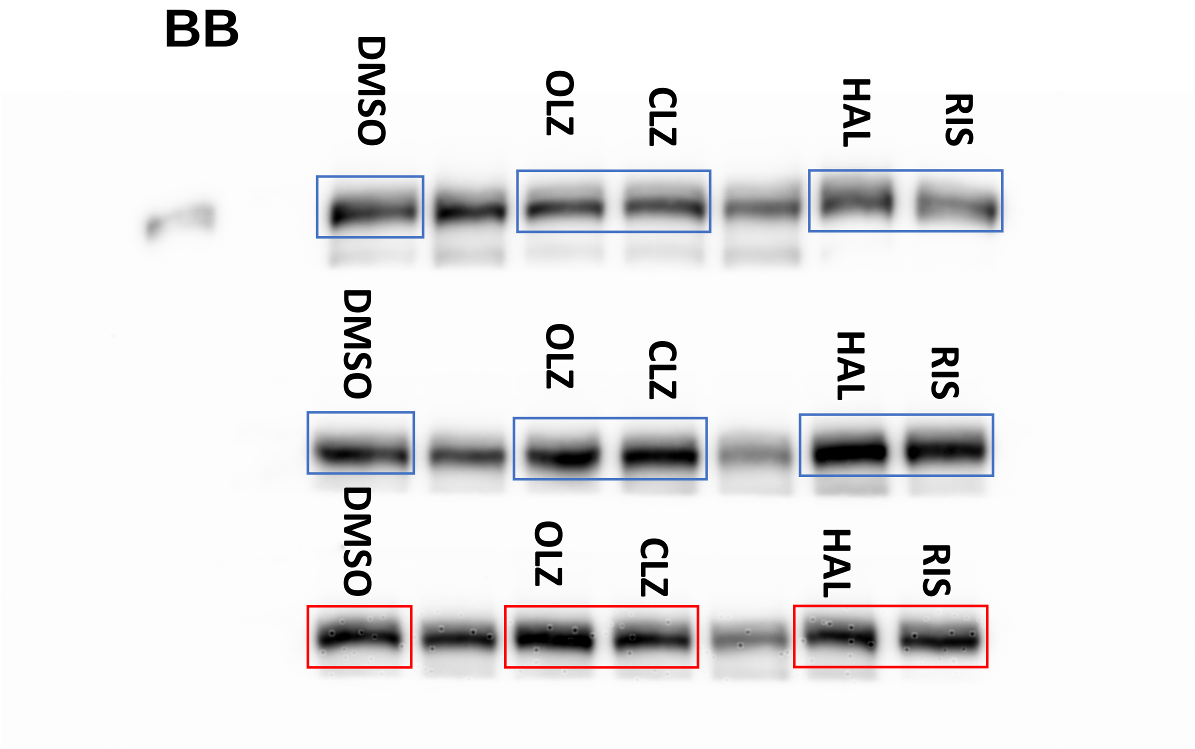

Figure S6BB. Original western blot images for ErbB1 (BB) used in Figure S2A. The parts surrounded by the red frames were used for the calculation and the display. The parts surrounded by the blue frames were used for the calculation. The values of the phosphorylated molecules (Figure S6AA) were divided by the values of the non-phosphorylated molecules(Figure S6BB).

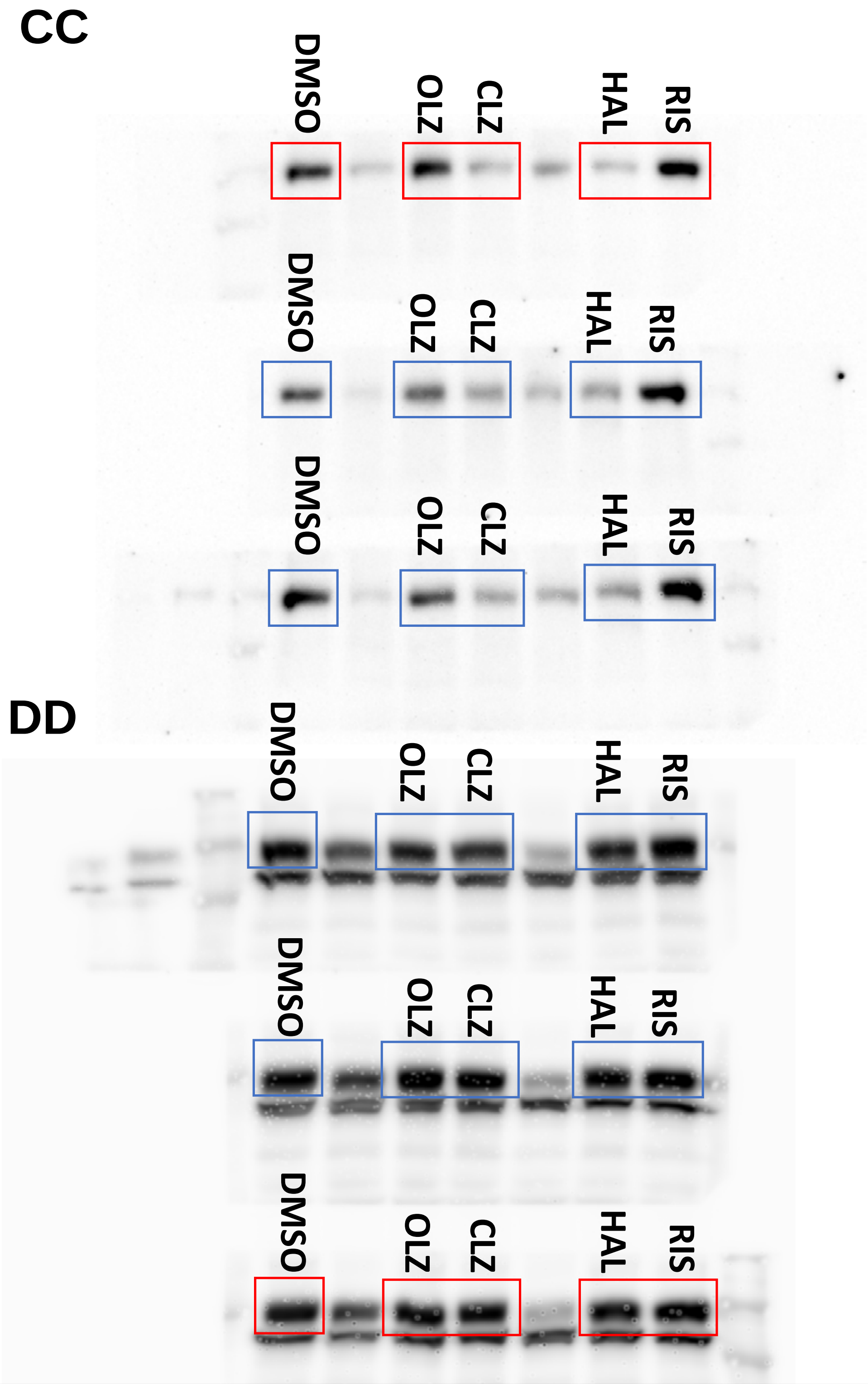

Figure S6CC, S6DD. Original western blot images for P-ErbB4 (CC) or ErbB4 (DD) used in Figure S2B. The parts surrounded by the red frames were used for the calculation and the display. The parts surrounded by the blue frames were used for the calculation. The values of the phosphorylated molecules were divided by the values of the non-phosphorylated molecules.

Figure S6-15

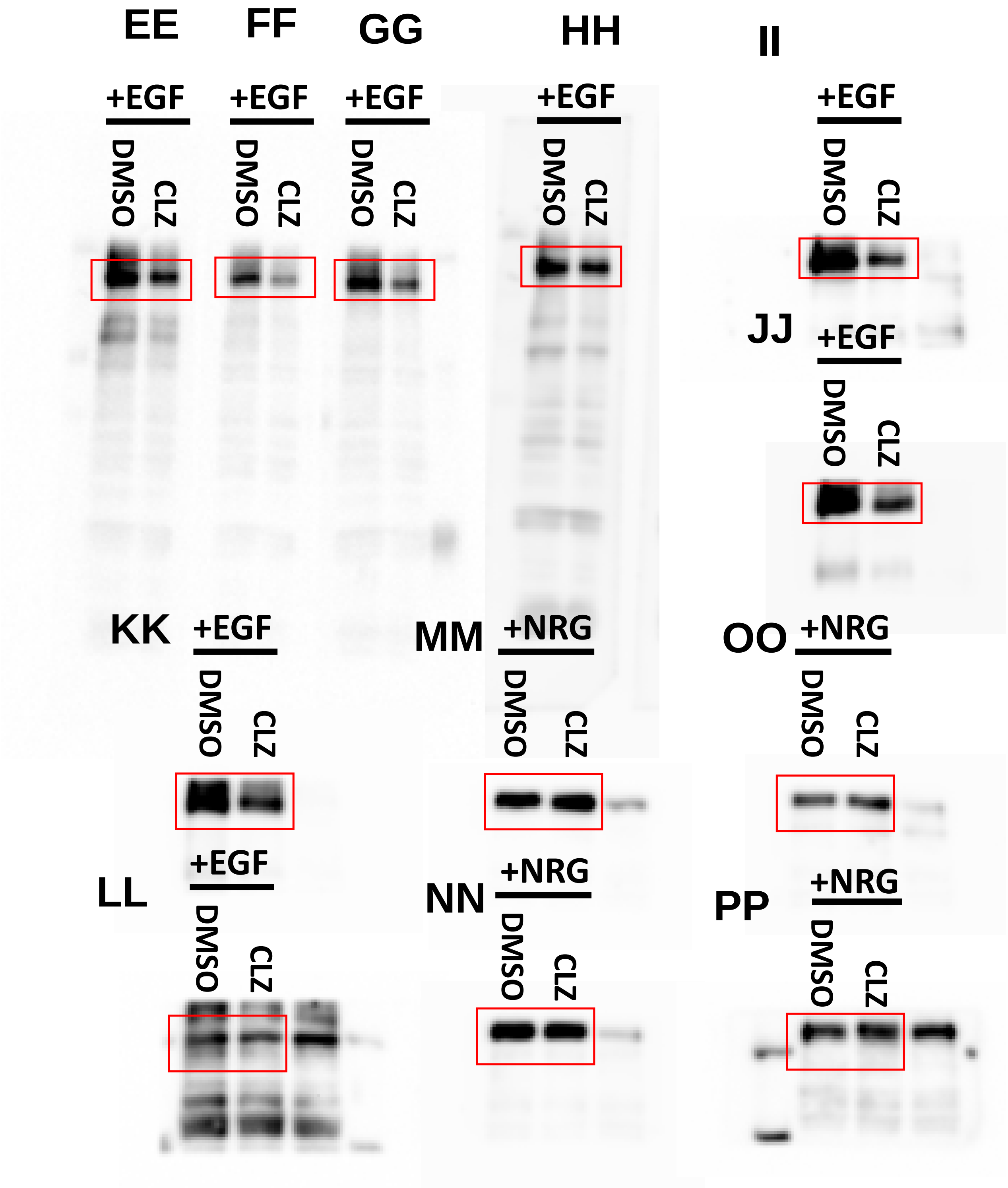

Figure S6EE-S6PP. Original western blot images for P-ErbB1(Tyr 845) (EE), P-ErbB1(Tyr 1045) (FF), P-ErbB1(Tyr 1173) (GG), ErbB1 (HH), P-ErbB2(Tyr 1139) (II), P-ErbB2(Tyr 1196) (JJ), P-ErbB2(Tyr 1248) (KK), ErbB2 (LL), P-ErbB3(Tyr 1197) (MM), P-ErbB3(Tyr 1289) (NN), P-ErbB3(Tyr 1328) (OO), or ErbB3(PP) used in Figure S3A, S3B, or S3C. The parts surrounded by the red frames were used for the display.

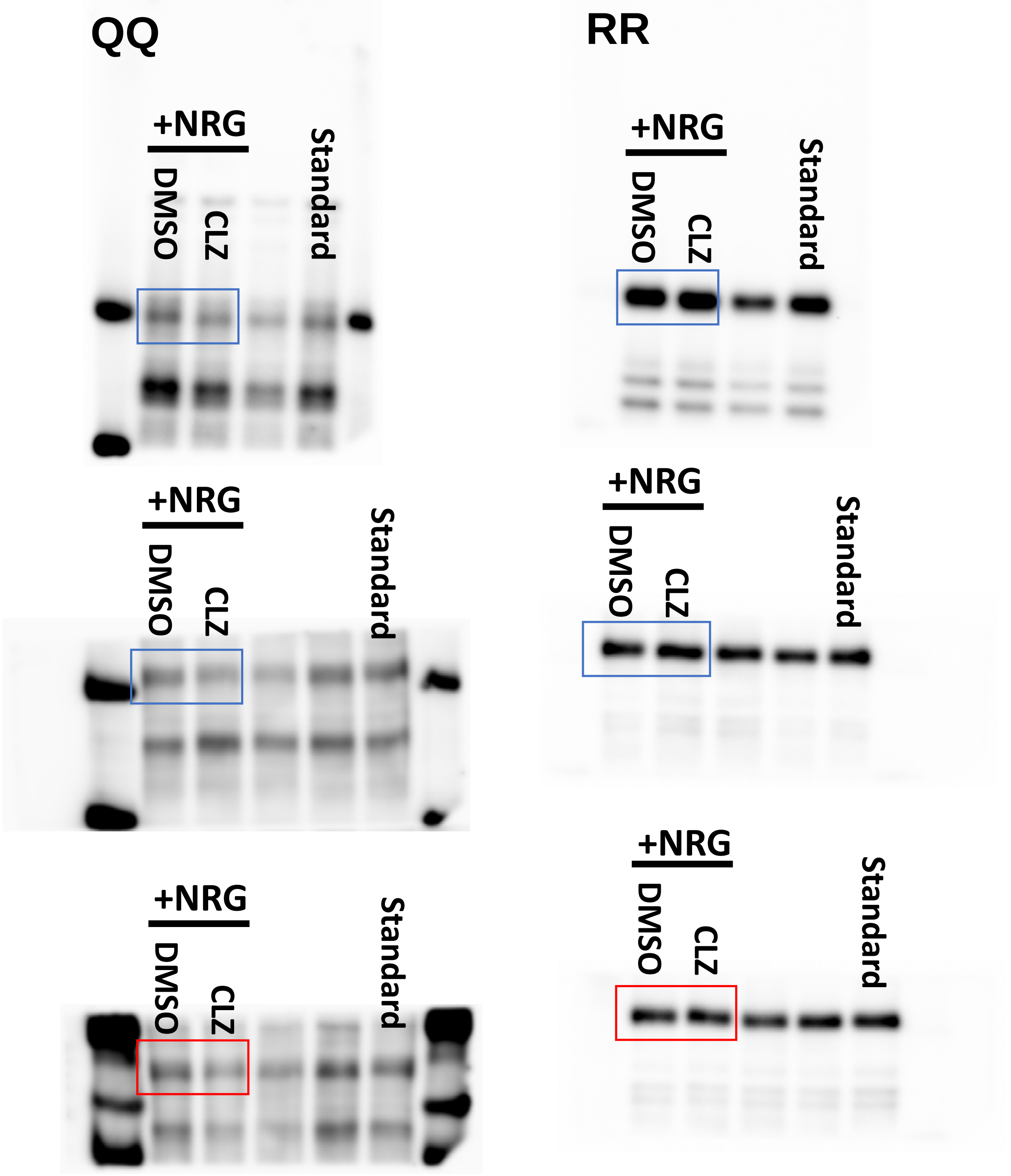

Figure S6QQ, S6RR. Original western blot images for P-ErbB4(Tyr 1056) (QQ) or ErbB4 (RR) used in Figure S3D. The parts surrounded by the red frames were used the display. The parts surrounded by the blue frames were used for the calculation.

Figure S6-17

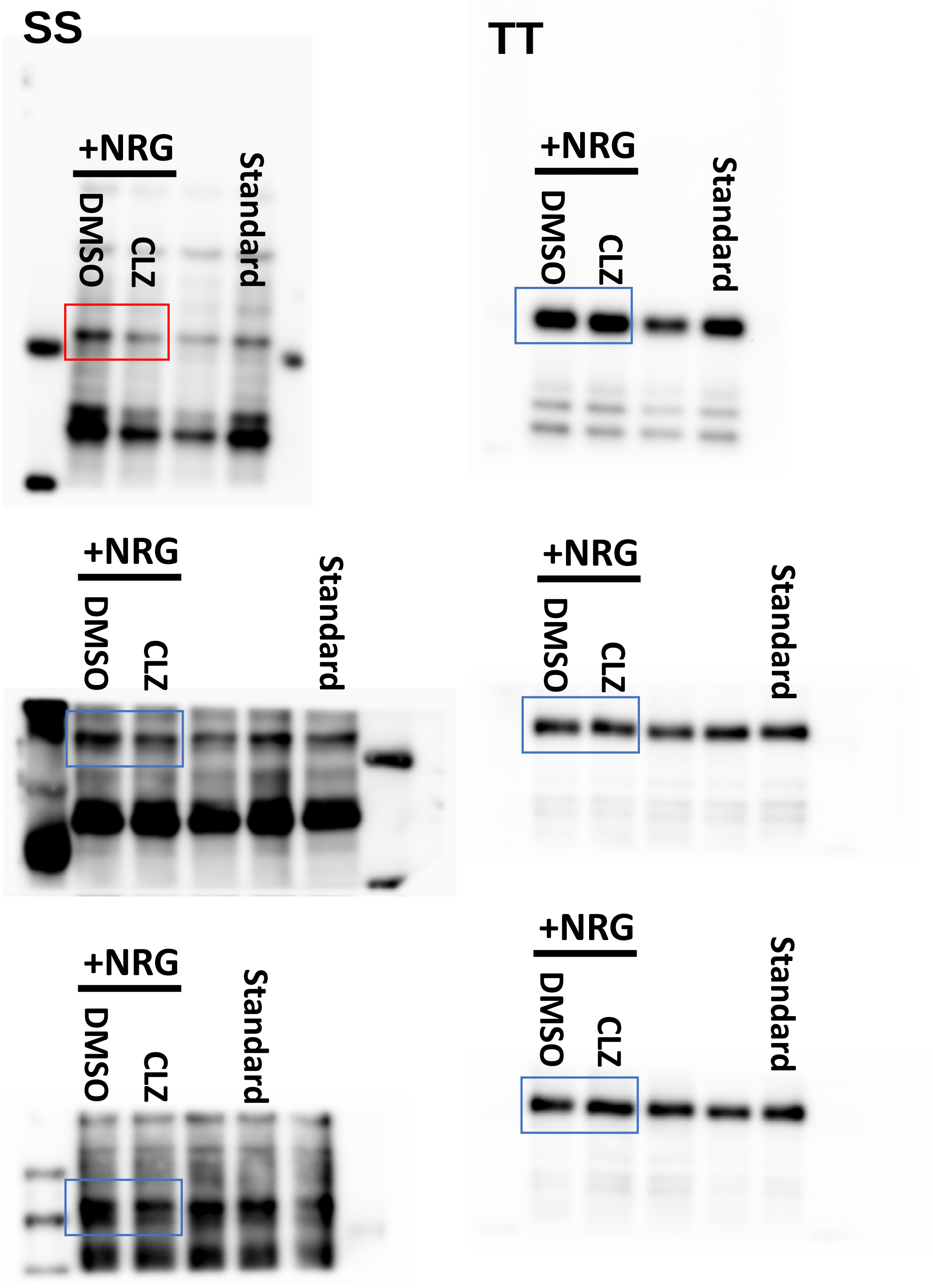

Figure S6SS, S6TT. Original western blot images for P-ErbB4(Tyr 1242) (SS) or ErbB4 (TT) used in Figure S3D. The parts surrounded by the red frames were used the display. The parts surrounded by the blue frames were used for the calculation.

Figure S6-18

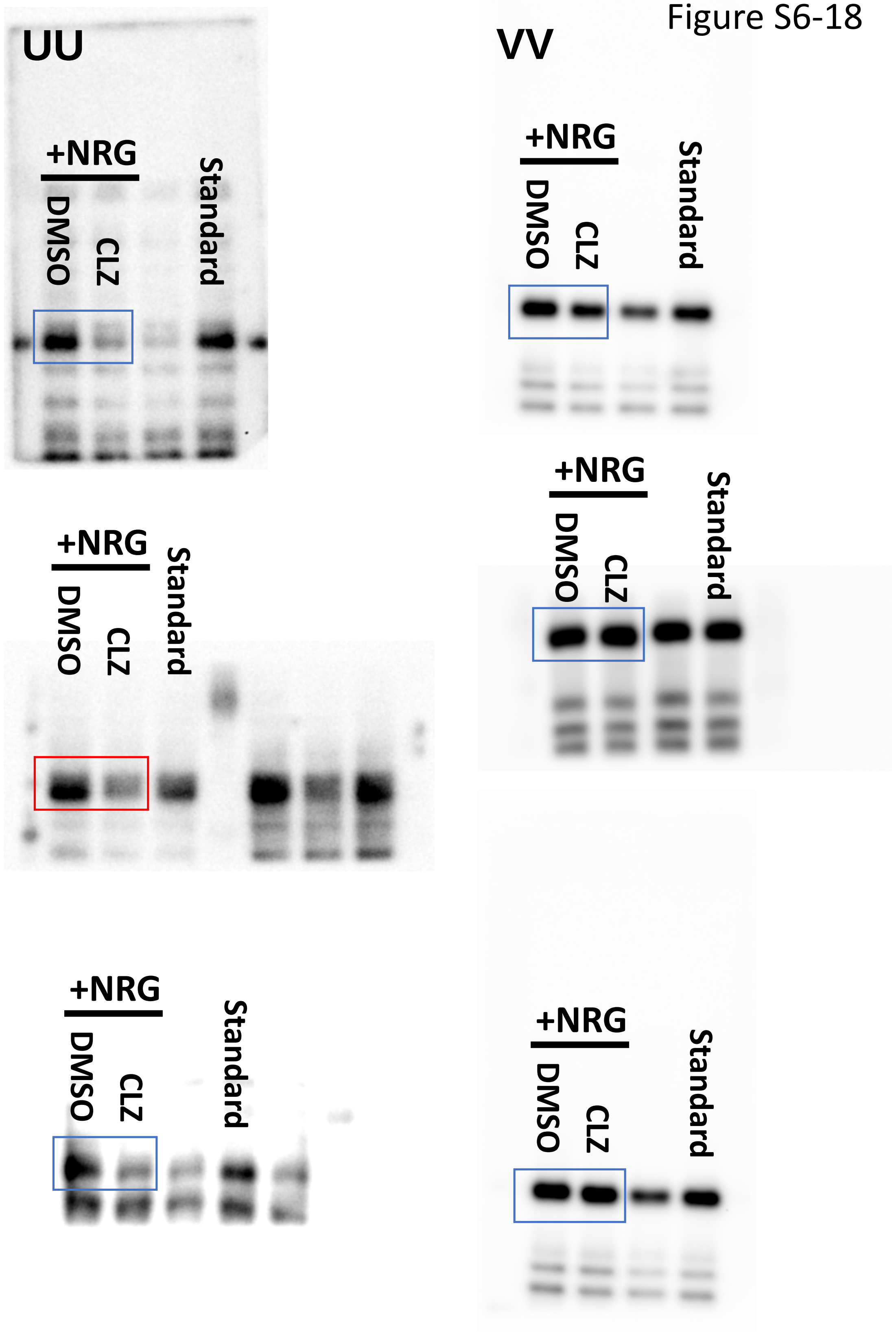

Figure S6UU, S6VV. Original western blot images for P-ErbB4(Tyr 1284) (UU) or ErbB4 (VV) used in Figure S3D. The parts surrounded by the red frames were used the display. The parts surrounded by the blue frames were used for the calculation.

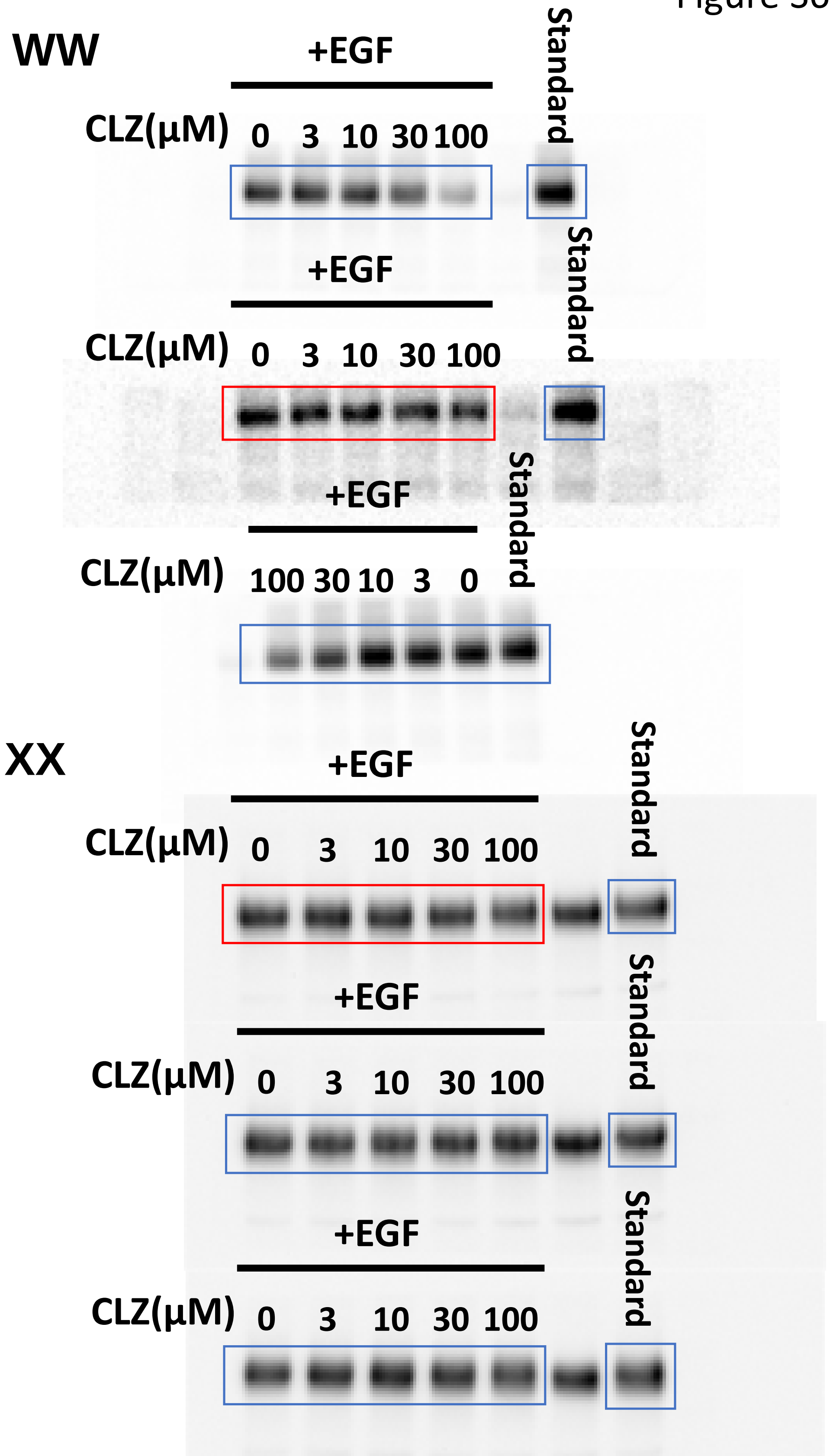

Figure S6WW, S6XX. Original western blot images for P-ErbB1 (WW) or ErbB1 (XX) used in Figure S4. The parts surrounded by the red frames were used for the calculation and the display. The parts surrounded by the blue frames were used for the calculation.

Figure S6-20

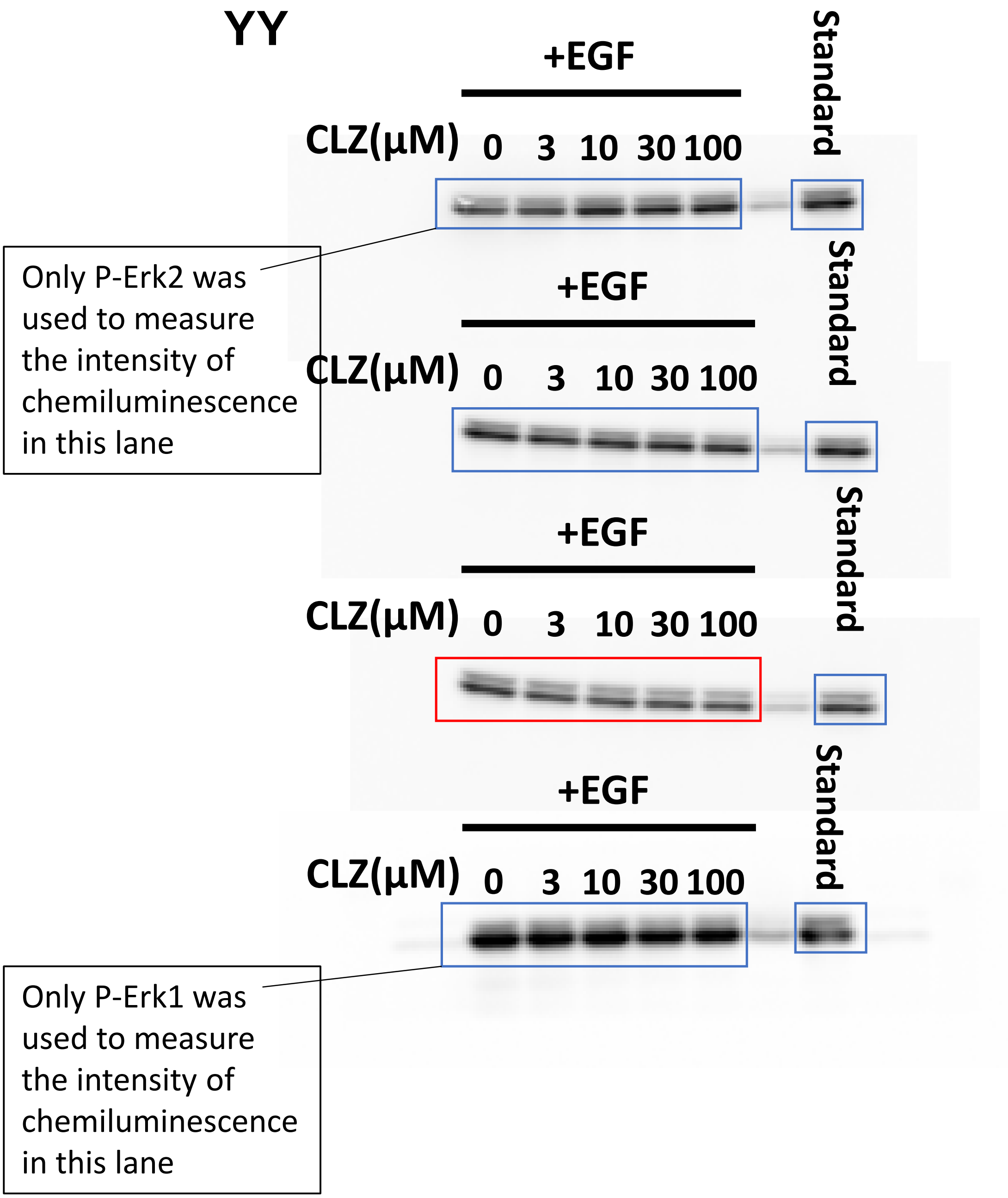

Figure S6YY. Original western blot images for P-Erk (YY) used in Figure S4. The part surrounded by the red frame was used for the calculation and the display. The parts surrounded by the blue frames were used for the calculation.

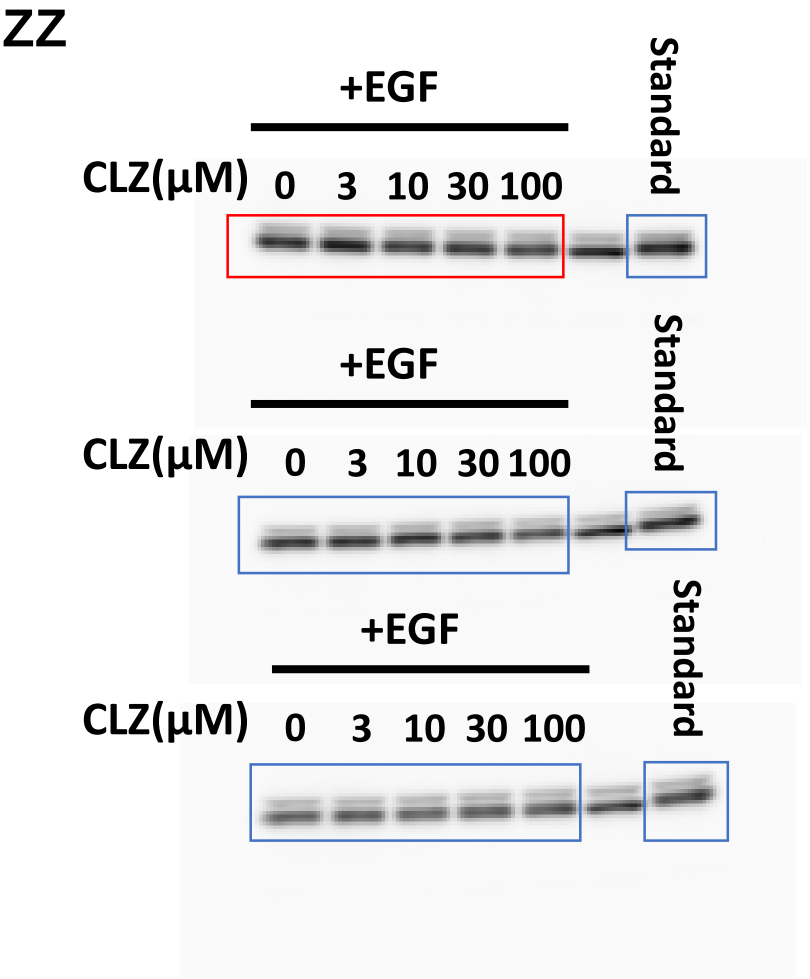

Figure S6ZZ. Original western blot images for Erk (ZZ) used in Figure S4. The part surrounded by the red frame was used for the calculation and the display. The parts surrounded by the blue frames were used for the calculation.

Figure S6-22

AAA

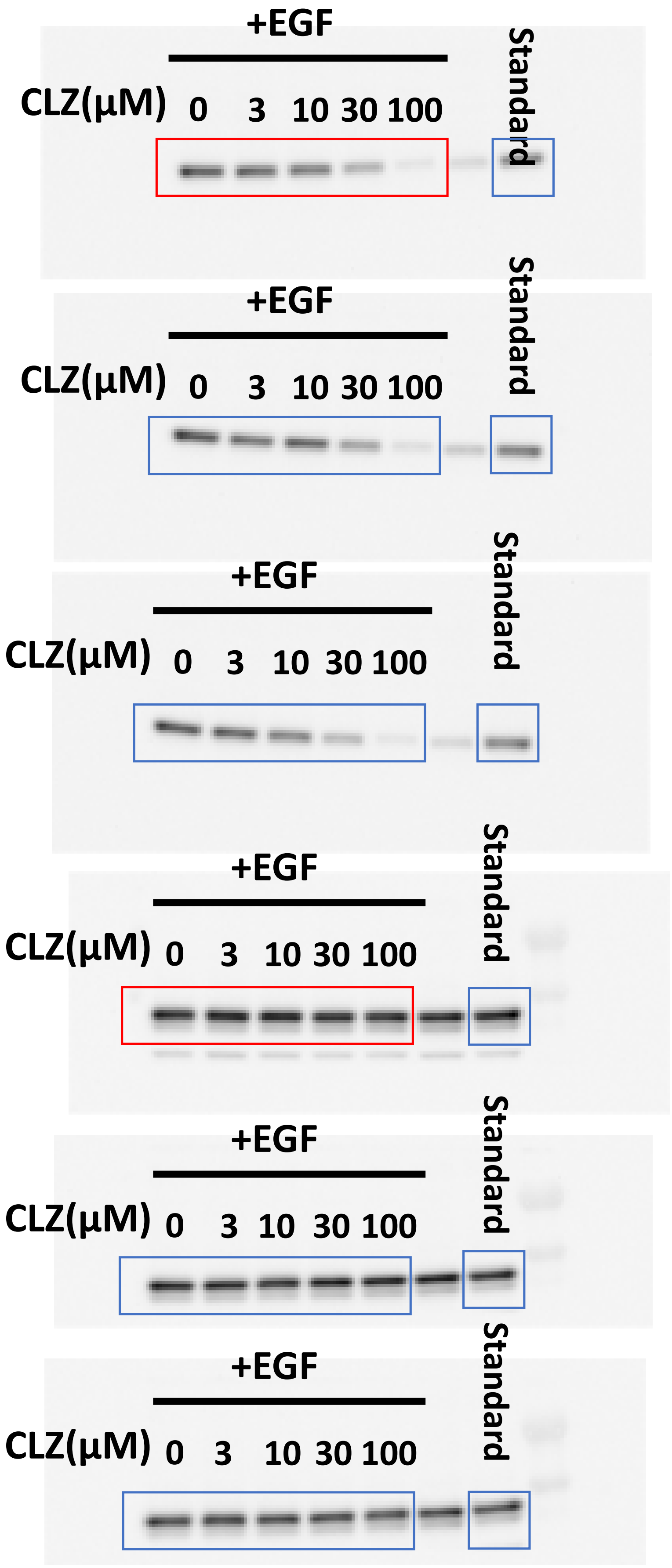

Figure S6AAA, S6BBB.
Original western blot images for P-Akt (AAA) or Akt (BBB) used in Figure S4.
The parts surrounded by the red frames were used for the calculation and the display. The parts surrounded by the blue frames were used for the calculation.

Figure S6-23

CCC

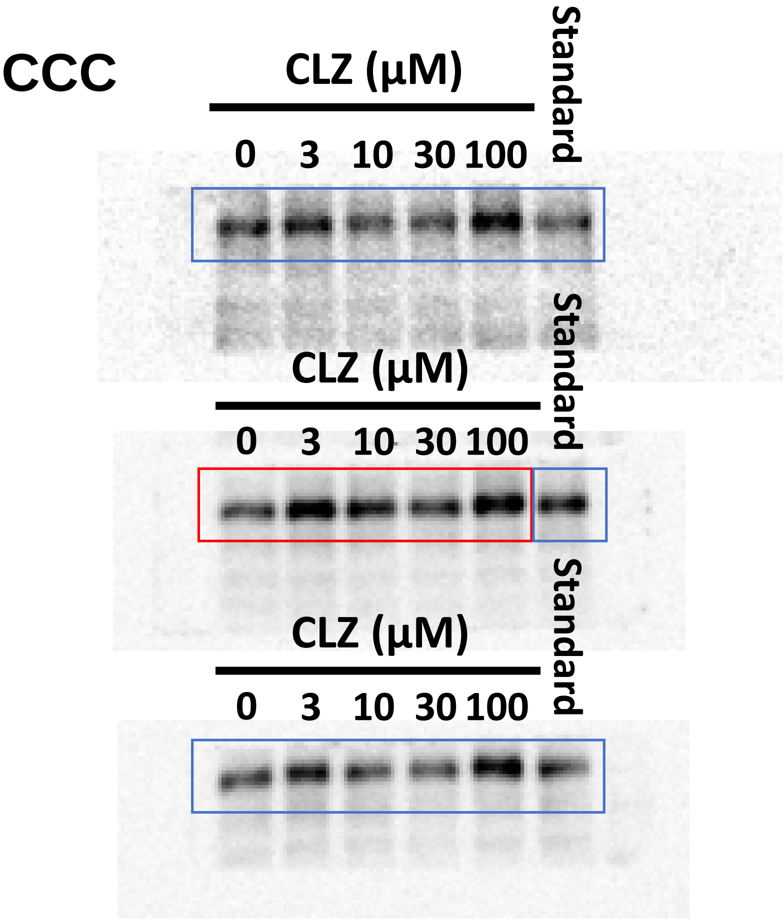

DDD

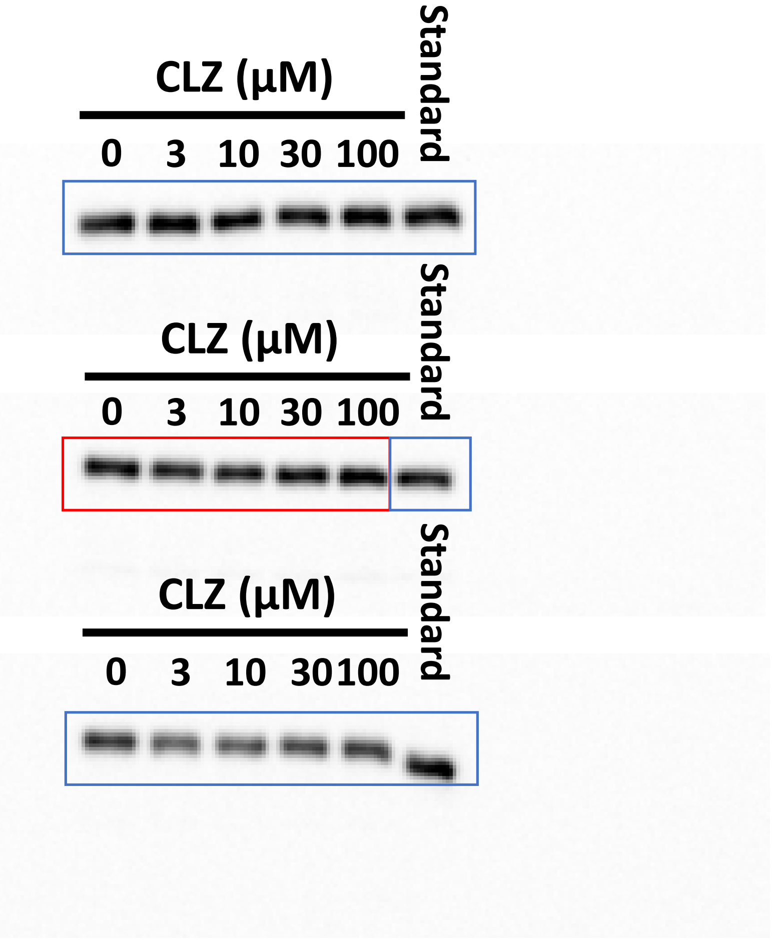

Figure S6CCC, S6DDD. Original western blot images for P-ErbB1 (CCC) or ErbB1 (DDD) used in Figure S5. The parts surrounded by the red frames were used for the calculation and the display. The parts surrounded by the blue frames were used for the calculation.
